# Microbiome and Skin Health: A Systematic Review of Nutraceutical Interventions, Disease Severity, Inflammation, and Gut Microbiota

**DOI:** 10.3390/microorganisms14010063

**Published:** 2025-12-26

**Authors:** Alia Ashkanani, Ghalya Ashkanani, Mahmoud Yousef, Mlaak Rob, Maha Al-Marri, Nesha Naseem, Sa’ad Laws, Ali Chaari

**Affiliations:** Weill Cornell Medicine Qatar, Qatar Foundation, Education City, Doha P.O. Box 24144, Qatar

**Keywords:** gut-skin axis, microbiome modulation, nutraceuticals, skin diseases, probiotics, prebiotics, synbiotics, dysbiosis, systematic review, atopic dermatitis, psoriasis, acne vulgaris, chronic urticaria, melasma

## Abstract

Skin disorders are a major global cause of morbidities, and increasing evidence links several to gut microbiome dysregulation. Because of this the bidirectional gut-skin axis, nutraceuticals have been proposed as therapeutic adjuncts, but their clinical effects across skin conditions remain unclear. To understand how pro/pre/synbiotics can affect health, we conducted a systematic review to investigate disease severity indices, inflammatory and immunological markers, quality of life, and changes in gut microbiota composition. PubMed, Embase, and Web of Science were utilized to identify relevant randomized clinical trials. Selected articles were pre-piloted for in-depth analysis and data extraction. We included 60 randomized controlled trials involving human participants with 5 dermatological conditions, including atopic dermatitis, psoriasis, acne vulgaris, chronic urticaria, and melasma, treated with probiotics, prebiotics, or synbiotics. Risk of bias was generally low across trials, with some having concerns. The SCORAD of the treated group was substantially lower than that of the placebo group in 30 of the 47 trials on atopic dermatitis. Inflammatory markers showed a range of results; some showed significant changes, while others produced contradictory results. Five trials that examined atopic dermatitis and psoriasis independently showed a significant improvement in Quality of Life. The PASI score was considerably lower in psoriasis in three of the five RCTs. Acne vulgaris, melasma, and chronic urticaria were not well documented. Major limitations included heterogeneity in interventions and outcomes, small sample sizes, and inconsistent reporting of microbiome analyses. Nutraceuticals show potential as additional treatments, but further, large scale studies are required.

## 1. Introduction

Skin and subcutaneous diseases include various inflammatory and immune-mediated conditions, including, but not limited to, atopic dermatitis (AD), psoriasis, acne vulgaris, chronic urticaria, and melasma. According to the Global Burden of Disease project, skin diseases are the fourth leading cause of nonfatal disease burden worldwide, compromising patients’ psychological well-being and quality of life [1,2,3,4]. Conventional therapeutic approaches often rely on prolonged use of immunosuppressive agents, which may lead to adverse effects and fail to address underlying microbial and systemic dysregulation [5,6]. Emerging research has highlighted the critical role of the gut microbiome in maintaining skin homeostasis via the gut-skin axis, suggesting its potential as a therapeutic target for dermatological conditions [7,8]. However, the evidence regarding the effectiveness of microbiome modulation in alleviating skin disease symptoms remains preliminary and requires further rigorous investigation.

The gut microbiome has emerged as a key factor in the pathophysiology of autoimmune and inflammatory skin conditions [7,8]. The gut microbiome is increasingly recognized as a contributor to diverse dermatologic conditions. While atopic dermatitis and psoriasis are primarily immune-mediated, others reviewed here, such as acne, chronic urticaria, and melasma, have more heterogeneous mechanisms involving inflammation, barrier dysfunction, and metabolic signaling. They share microbiome-related features, including dysbiosis-driven inflammation, altered microbial metabolites, and impaired epithelial barrier function, suggesting partially convergent pathways despite distinct clinical presentations. Through the fermentation of non-digestible dietary fibers, gut microbes produce short-chain fatty acids (SCFAs), which support cardiometabolic health, vitamin synthesis, and immune regulation [9]. In a healthy state, the gut microbiota and host exist in a stable symbiotic relationship maintained by physical, chemical, and immunological barriers [10]. Disruption of this balance is known as gut dysbiosis, and leads to microbial imbalance, systemic inflammation, oxidative stress, and exacerbation of chronic disease [11,12,13]. We define dysbiosis as disruption in the normal composition, diversity, or balance of the microbial community compared with a healthy state. Due to its extensive crosstalk with other organ systems, gut dysbiosis is increasingly recognized as a contributing factor to inflammatory skin diseases via the gut-skin axis. The skin, like the gut, hosts its own microbiome and shares functional similarities, including barrier defense and immune signaling. Studies have shown that gut-derived metabolites could potentially influence skin physiology and immune responses, with imbalances in gut microbiota perhaps contributing to various skin disorders [14,15].

Dysbiosis has been proposed to contribute to atopic dermatitis, psoriasis, acne vulgaris, chronic urticaria, and melasma, with each condition exhibiting distinct gut microbial signature patterns [11,16]. In AD, reduced levels of anti-inflammatory SCFAs such as acetate, butyrate, and propionate impair T-reg cell activation and allow Th2-driven inflammation through cytokines like IL-4, IL-5, and IL-13 [17,18]. This may explain increased intestinal permeability and microbial translocation. Similarly, patients with acne vulgaris often show a decrease in microbial diversity, specifically in *Clostridiales*, *Lachnospiraceae*, *Ruminococcaceae*, and *Clostridia*. This is associated with immunological dysfunction and systemic inflammation. Clinical improvements in acne have also been associated with shifts in gut microbial composition, with reductions in dysbiosis-linked inflammation paralleling improvements in disease severity. Chronic urticaria, an immune-mediated condition characterized by histamine release and Th1/Th2 cytokine activity, has also been linked to dysbiosis marked by increased opportunistic pathogens and reduced beneficial bacteria. Altered gut metabolites such as increased SCFAs and decreased unsaturated fatty acids and LPS disrupt T-reg cell development, fueling inflammation via IL-4, IL-17, IL-3, and TNF-α; probiotic strains like *Lactobacillus* and *Bifidobacterium* may mitigate these effects [19,20]. Melasma, a hormonally influenced pigmentary disorder, has been associated with disruptions in estrogen metabolism due to the loss of estrogen-metabolizing bacteria such as Actinobacteria [12,21]. Studies by Liu et al. [21] and Yu and Wu support that lower levels of these bacteria may elevate circulating estrogen, perhaps worsening melasma severity and highlighting the gut microbiome’s role in hormone-regulated dermatoses [22,23].

Standard first-line treatments for inflammatory skin conditions include topical corticosteroids, antihistamines, emollients, and immunomodulating medications [5]. However, these mediations often require chronic usage to sustain efficacy and may cause life-threatening side effects such as inflammatory rashes, drug-induced vasculitis, and neutrophilic dermatoses [5,6]. Given the chronic nature of these conditions and the limitations of current treatments, there is a growing need for novel therapeutic options that address underlying pathophysiology to prevent disease progression and improve patients’ quality of life [24].

Nutraceutical interventions such as probiotics, prebiotics, and synbiotics have been proposed to improve gut barrier integrity, restore microbial balance, and suppress inflammatory cytokines [25,26,27]. Resultantly, they have been suggested to decrease disease severity assessment indices and improve patients’ quality of life [28,29,30,31]. Probiotics are live microorganisms that, when taken in adequate amounts, provide health benefits to the host. Common strains include *Lactobacillus* (e.g., *L. rhamnosus*, *L. acidophilus*), *Bifidobacterium* (e.g., *B. longum*), and the yeast *Saccharomyces boulardii*. They support gut health by enhancing barrier function, competing with pathogens, producing beneficial metabolites like SCFAs, and modulating immune responses. Prebiotics are non-digestible food components that selectively promote the growth of beneficial gut bacteria. It is hypothesized that SCFAs exert their immunomodulatory and epithelial effects through several well-defined pathways, including activation of GPR41 and GPR43, inhibition of histone deacetylases (HDACs), and modulation of Toll-like receptor (TLR) signaling, each of which contributes to reduced inflammatory cytokine production and potentially improves barrier integrity. They have been demonstrated to increase SCFA production, enhance the Th1/Th2 balance, boost lymphocyte and leukocyte levels in GALT, and increase intestinal IgA secretion [32]. To qualify as prebiotics, they must resist digestion in the upper GI tract, be fermented by gut microbes, and promote health-associated bacterial populations. They can increase SCFA production, improve gut barrier integrity, and enhance overall microbiome diversity. These immunomodulatory effects help maintain intestinal and systemic immune homeostasis. Finally, synbiotics are products that combine probiotics and prebiotics to maximize their benefits. The prebiotic component serves as a food source for the probiotic strains, improving their survival, colonization, and activity in the gut. This synergistic approach could enhance microbiota balance, immune modulation, and gastrointestinal function more effectively than either component alone.

The current literature remains unclear due to variations in study design, outcome measures, and intervention durations, highlighting the need for a systematic review. This systematic review aims to critically evaluate the effectiveness of nutraceuticals in managing inflammatory skin diseases. By evaluating their impact on disease severity scores, inflammatory cytokine profiles, gut microbiota composition, and patient-reported outcomes, this systematic review seeks to clarify the therapeutic potential of targeting the gut-skin axis. Our findings are intended to inform clinical practice and support the development of targeted nutraceuticals for the long-term, non-immunosuppressive management of skin inflammation. Building on previous contributions that focused on individual conditions [29,33,34,35] this systematic review incorporates newly published trials and broadens the scope to include five dermatological conditions: AD, psoriasis, acne vulgaris, chronic urticaria, and melasma. Our study aims to critically evaluate the effectiveness of nutraceuticals in the management of inflammatory skin diseases.

Despite their distinct clinical manifestations, atopic dermatitis, psoriasis, chronic urticaria, acne vulgaris, and melasma share common upstream disruptions involving gut microbiota imbalance, including reduced microbial diversity, impaired intestinal barrier function, and decreased production of SCFAs. These disturbances have been increasingly linked to systemic inflammation and immune dysregulation, positioning the gut-skin axis as a potential therapeutic target across dermatological conditions. Given these shared microbial signatures, our review examines whether nutraceuticals, specifically probiotics, prebiotics, and synbiotics, can serve as a unifying therapeutic strategy across these diseases. By synthesizing evidence across conditions, we aim to identify both overarching mechanisms of action and disease-specific responses, providing a nuanced understanding of when and how these interventions may be most effective.

## 2. Methods

### 2.1. Study Protocol and Literature Search

This systematic review adheres to the reporting standards outlined in the Preferred Reporting Items for Systematic Reviews and Meta-analysis (PRISMA) [36]. We searched for both non-grey literature on PubMed, Web of Science, and Embase databases (latest search 10 December 2025). We also searched grey literature (conference proceedings) on Web of Science. Detailed search strategies for each database are provided in the Supplementary Materials. This review was not registered on PROSPERO. A meta-analysis was not performed due to substantial clinical and methodological heterogeneity across the included studies. The trials differed markedly in patient populations, probiotic/prebiotic/synbiotic formulations, dosing strategies, duration of treatment, and outcome measures. Additionally, several studies reported results using non-comparable disease severity indices, inconsistent inflammatory biomarkers, or incomplete quantitative data, which precluded pooling of effect estimates.

### 2.2. Inclusion and Exclusion Criteria

Our selection strategy aimed to identify studies providing clinical evidence on the effects of nutraceuticals on dermatological outcomes. We included clinical trials that investigated the effects of probiotics, prebiotics, and synbiotics on skin health. To be considered eligible, studies were required to include both intervention and control groups and to assess at least one of the following outcomes: disease severity indices; inflammatory or immunological markers; quality of life (measured using Quality of Life (QoL) questionnaires including the Dermatology Life Quality Index (DLQI), Children’s Dermatology Life Quality Index (CDLQI), and Infants’ Dermatitis Quality of Life Index (IDQoL)); or gut microbiome composition in patients with inflammatory skin diseases. We did not apply any restrictions on follow-up duration, participant age, sex, ethnicity, country of origin, and year of publication. We excluded reviews, conference proceedings, abstracts, editorials, animal studies, non-clinical literature, single-arm trials, case–control, and cohort studies. We restricted inclusion to interventional clinical trials because they provide the highest level of evidence for evaluating treatment efficacy and minimizing confounding, whereas observational studies cannot reliably determine causal effects for this research question. We excluded studies that did not primarily evaluate nutraceuticals or were published in languages other than English. We excluded studies with wrong outcomes, wrong study design, full text not being found, or wrong interventions.

These skin diseases were studied together because they share common pathogenic features involving the microbiome-skin axis, immune dysregulation, and systemic inflammation. Probiotics, prebiotics, and synbiotics have been explored as adjunctive treatments, offering a unifying therapeutic rationale. Grouping them allows comparison of general versus condition-specific benefits to guide targeted recommendations.

These studies were assessed for eligibility, resulting in the exclusion of 94 studies for reasons such as wrong outcomes [15], wrong study design [37], and other criteria like full text not being found [17] or wrong interventions [26].

### 2.3. Screening and Data Extraction

After removing duplicate records, we screened the remaining articles based on their titles and abstracts. Full texts of potentially relevant studies were then reviewed to determine eligibility. Two reviewers independently conducted the screening process, and any discrepancies were resolved through mutual consensus. For data extraction, we used a standardized spreadsheet to systematically collect relevant information from each included study. Extracted data included author names, country, study design, population demographics, and intervention characteristics. All tabulation and visualization of data was conducted using Microsoft Excel version 16.104 (Microsoft Corporation, Redmond, WA, USA) and Zotero reference management software version 7.0 (Corporation for Digital Scholarship, Vienna, VA, USA).

### 2.4. Risk of Bias Assessment

2 reviewers independently assessed study quality using the conservative Risk of Bias 2 (RoB 2). This tool examines 5 domains: D1 (randomization), D2 (adherence to interventions), D3 (missing data), D4 (outcome measurement), and D5 (reporting). Each study received a rating of low, some concerns, or high risk of bias, ensuring a consistent assessment of study quality.

## 3. Results

### 3.1. Study Characteristics and Risk of Bias

**Figure 1:**
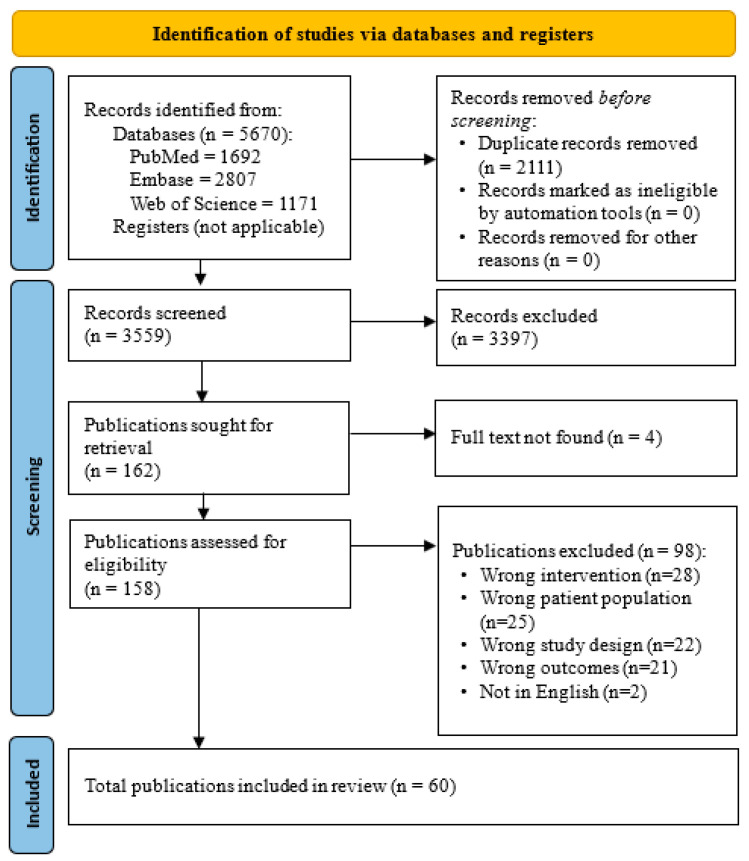
PRISMA Flowchart of Study Selection Process.

A total of 5670 records from databases including PubMed, Embase, and Web of Science (Figure 1). After the removal of 2111 duplicates, 3559 studies proceeded to the title and abstract screening phase. Of these, 3397 were excluded based on relevance to the review criteria. Four studies could not be retrieved successfully leaving a total of 158 studies that underwent full text screening. 60 studies met the inclusion criteria and were included in the review. Conference proceedings were screened but excluded due to insufficient methodological and outcome data. Out of the total studies screened, 98 were excluded for various reasons. Of these, 28 studies were excluded due to the wrong intervention, 25 because of an incorrect population, 22 for having the wrong study design, and 21 for reporting the wrong outcomes. Additionally, 2 studies were not in English. Inter-rater agreement was evaluated using Cohen’s Kappa to assess the consistency between reviewers. The overall Cohen’s Kappa for all reviewers in title/abstract was 0.743. Pairwise analysis for full text screening demonstrated a Kappa of 0.829 between Reviewer 1 and Reviewer 2.

The included studies encompassed a total sample size of 3732 participants, with 2104 assigned to intervention groups and 1769 to control groups. The highest number of studies originated from Japan (n =7, followed by Iran (n = 6), Italy (n = 5), Finland and Spain (n = 4 each), and Taiwan (n = 3). The median year of publication was 2016, ranging from 2000 to 2024 and an interquartile range (IQR) of 2010 to 2021.

The 60 included studies focused on various skin and subcutaneous diseases and assessed the use of different nutraceuticals, including probiotics, prebiotics, and synbiotics. Of these, 47 studies focused on atopic dermatitis (43/47 probiotics, 2/47 prebiotics, 3/47 synbiotics), 5 on psoriasis (4/5 probiotics, 1/5 synbiotics), 5 on acne vulgaris (5/5 probiotics), 2 on chronic urticaria (1/2 probiotics, 1/2 synbiotics), and 1 on melasma (1/1 synbiotics).

The median study duration was 12 weeks (reported in 22 studies), with an IQR of 4 weeks and a maximum duration of 22 weeks. Interventions included probiotics (52 studies), prebiotics (2 studies), and synbiotics (6 studies), with *Lactobacillus rhamnosus* being the most used probiotic strain (13 studies). The median dosage for was 10 billion CFU/day for probiotics (range: 19 billion CFU/day, IQR: 7.5 billion CFU/day), 1 g for prebiotic (range: 1.5 g, IQR: 0.75 g), and 1.65 billion CFU/day for synbiotics (range: 6 billion CFU, IQR: 2.025 billion CFU). The mean age of participants across studies was 13.98 years, and the overall male-to-female ratio was approximately 1.47, indicating a predominance of male participants.

**Figure 2:**
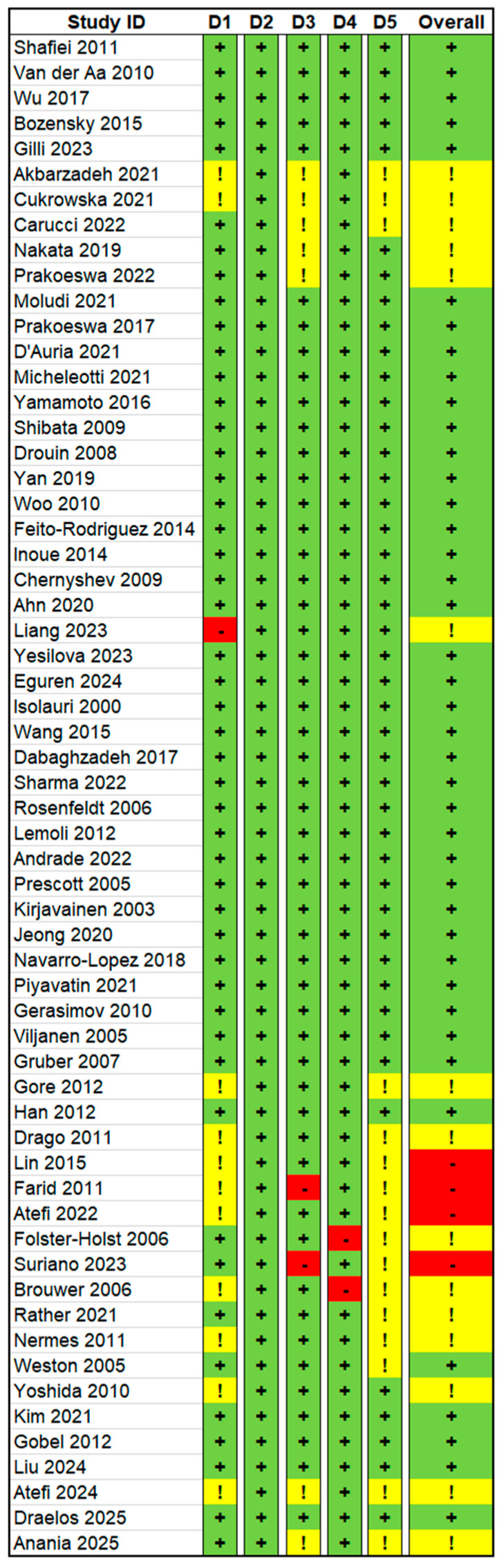
Risk of Bias Assessment for Randomized Controlled Trials (RCTs). Four RCTs were rated as high risk of bias (red), fifteen RCTs showed some concerns (yellow), and the remaining RCTs were rated as low risk of bias (green).

Risk of bias assessment indicated that the majority of studies (n = 41, 68.3%) were rated as having a low risk of bias across all domains (Figure 2). Fifteen studies (25.0%) were judged to have some concerns in at least one domain, most frequently in D1 or D3 (randomization or deviation from intended interventions). Of these, 5 of 15 demonstrated improvements in their respective clinical outcomes. Carucci et al., 2022 and Prakoeswa et al., 2022 reported improvements in SCORAD scores, while Akbarzadeh et al., 2022, Liang et al., 2024, and Atefi et al., 2025 showed improvements in PASI, TLC, and GAGS scores, respectively [38,39,40,41,42]. The remaining 10 studies reported no significant improvements in their clinical outcomes, like SCORAD or incidence of atopic dermatitis [26,38,39,40,41,42,43,44,45,46,47,48,49,50,51].

Four studies (6.7%) were rated as having a high risk of bias in D4 and D5 (outcome measurement or reporting), with 3 of 4 showing improvements in respective clinical outcomes. Atefi et al., 2022 showed enhancements in both disease activity and quality of life, Lin et al., 2015 and Farid et al., 2011 reported improved SCORAD scores, while Suriano et al., 2023 found no improvement in PASI scores [52,53,54,55].

Across the included studies, disease severity was frequently measured using validated, condition-specific instruments such as SCORAD for atopic dermatitis, PASI for psoriasis, GAGS or lesion counts for acne, UAS7 for chronic urticaria, and mMASI for melasma.

### 3.2. Atopic Dermatitis

#### 3.2.1. Effect of Microbiome-Targeted Therapies on Disease Severity Index

47 studies evaluated AD severity using SCORAD to evaluate the effects of probiotics, prebiotics, and synbiotics (Table 1). Of these, studies demonstrated statistically significant SCORAD reductions in intervention groups versus placebo, and 17 studies showed no statistically significant difference despite improvements in both groups. 4 studies either had insufficient or missing data regarding *p*-values or minimal SCORAD reductions in both groups. One study, Liu et al. 2025, studied FMT and observed markedly better clinical improvement than placebo, with significantly more patients achieving EASI-50 (84.6% vs. 25% at week 4, *p* = 0.0176; 92.3% vs. 37.5% at week 8, *p* = 0.0139) [56]. There were no significant differences between groups in SCORAD, IGA, ADCT, DLQI, VAS, POEM, HADS, or adverse events.

#### 3.2.2. Effect of Nutraceuticals on Inflammatory and/Immunological Markers

19 RCTs investigating the effects of nutraceuticals on inflammatory and immunological markers in AD patients were identified with mixed results (Table 1).

Two trials reported significant reductions in IgE levels: Nakata et al. observed a Δ = −0.14 with *Lactobacillus acidophilus* L-92, and Yeşilova et al. found a reduction of 145.1 IU/mL (*p* = 0.0035) [44,72]. However, Yan et al. [66] reported a significant increase in IgE levels in the intervention group (Δ = +61.88, *p* = 0.038) compared to baseline, while the rest of the studies, like Ahn [71], Van der Aa et al. [58], Prakoeswa et al. [61], Prakoeswa et al. [39], Andrade et al. [78], Shibata et al. [65], and Cukrowska et al. [43], Also, Han et al. [86] and Gøbel et al. [88] showed mostly nonsignificant changes within each group and between intervention and placebo groups.

Seven studies investigated eosinophils counts with variable results (Table 1). Among these, one study reported statistically significant between-group differences, while the others showed either within-group changes or nonsignificant results [56]. Inoue et al. [69] showed significant reduction between intervention and placebo groups (*p* < 0.05) following 8-week supplementation with *Bifidobacterium breve* though specific counts were not reported.

Five studies assessed levels of IL-4 in patients with AD (Table 1), with significant reductions observed in intervention groups of Prakoeswa et al. [61] and Prakoeswa et al. [39], (Δ = 6.007, intervention; Δ = 4.197, placebo, *p* = 0.000, Δ = 2.906, intervention; Δ = 0.651, placebo, *p* < 0.05), respectively. Han et al. [86] (*p* > 0.05), Farid et al. [54] (*p* = 0.3) and Rosenfeldt et al. [76] (*p* = 0.35) showed no significant changes between intervention and placebo groups.

IFN-γ was investigated in 7 RCTs (Table 1). Of these, 5 trials found significant changes. Han et al. [86], Prakoeswa et al. [61], Yeşilova et al. [72] and Rosenfeldt et al. [76] reported a significant reduction in IFN-γ levels between intervention and placebo (*p* < 0.05, *p* = 0.006, *p* = 0.0011, *p* = 0.04, respectively) as well as significant changes within placebo and intervention groups, while Prakoeswa et al. [39] found a significant increase (*p* < 0.05) and Farid et al. [54] found no significant change between intervention and placebo groups (*p* = 0.7).

IL-10 was evaluated by 2 studies (Table 1). Rosenfeldt et al. demonstrated that supplementation with *Lactobacillus rhamnosus* 19070-2 and *Lactobacillus reuteri* DSM 122460 (10^10^ CFU of each strain twice daily) for 6 weeks versus the placebo resulted in no significant change between the two intervention groups [76]. Similarly, Gobel et al. observed reductions with *Lactobacillus acidophilus* and *Bifidobacterium animalis* (Δ: 47.5 and 159.7, respectively), neither showed significant changes compared to control groups [88].

Two trials investigated the impact of nutraceuticals on IL-31 levels (Table 1). Jeong et al. reported that IL-31 levels increased in the placebo group while significantly decreasing in the intervention group receiving probiotic supplementation (Δ: −599.86 ± 985.95 pg/mL, *p* = 0.0431) [81]. This contrasted with the placebo group, which showed an increase of 330.28 ± 1038.98 pg/mL. The significant reduction in the intervention group highlights the potential of synbiotics in modulating IL-31 levels. In contrast, Gobel et al. observed mixed results across different interventions [88]. IL-31 levels increased in both the placebo group (Δ: increase from 133.3 to 193.3 pg/mL; *p* = 0.25) and the group receiving *Bifidobacterium animalis* supplementation (increase from 71.0 to 105.8 pg/mL; *p* = 0.34). However, supplementation with *Lactobacillus acidophilus* led to a minimal decrease (49.1 to 49.0 pg/mL; *p* = 0.36). None of the changes reported by Gobel et al. reached statistical significance.

Two RCTs investigated the effect of interventions on Thymus and Activation-Regulated Chemokine (TARC) levels (Table 1). Nakata et al. reported a significant reduction in TARC levels in the intervention group compared to control, with a mean change of −504 pg/mL (range: −19,279 to 1068; *p* = 0.03) [44]. The control group exhibited a change of 86 pg/mL (range: −29,661 to 805), which was not statistically significant. Similarly, Michelotti et al. observed a significant decrease in TARC levels following the intervention [63]. Among the intervention group, the mean change was 1.5 ± 0.22 pg/mL, which was significant compared to both baseline and the control group (*p* = 0.001). In contrast, the control group experienced a smaller reduction of 0.4 ± 0.14 pg/mL, which was not significant.

Further, two RCTs evaluated the effect of interventions on CCL17/TARC levels (Table 1). Yan et al. reported a significant reduction in CCL17/TARC levels in the intervention group compared to baseline, with levels decreasing from 186.58 ± 364.84 to 133.94 ± 270.42 (*p* = 0.017) [66]. The control group showed a similar reduction from 220.64 ± 385.42 to 128.37 ± 238.53, but the difference in change between the intervention and control groups was not statistically significant (*p* = 0.990). Woo et al. also observed a significant reduction in CCL17/TARC levels in the intervention group compared to placebo groups (*p* = 0.03), with levels decreasing from 2.60 ± 0.10 to 2.27 ± 0.13. Conversely, the control group showed a smaller and nonsignificant reduction from 2.50 ± 0.07 to 2.48 ± 0.07 [67].

Each of the following cytokines and immune markers were investigated by 1 RCT (Table 1). Prakoeswa et al. found significant reductions in IL-17 levels (*p* < 0.05) and significant increases in Foxp3 levels (*p* < 0.05) in the intervention group compared to smaller changes in the placebo group [39]. Michelotti et al. reported significant decreases in TNF-α (*p* = 0.001) and thymic stromal lymphopoietin (TSLP) (*p* = 0.001) levels in the intervention group, while the placebo group showed non-significant reductions [63]. Yeşilova et al. [72] observed significant reductions in IL-5 (*p* = 0.0012) and IL-6 (*p* = 0.0016) in the intervention group, contrasting with increases in the placebo group. Another study found a significant reduction in TGF-β levels (*p* < 0.05) in the intervention group compared to placebo [69]. Rosenfeldt et al. reported reductions in IL-2 in both groups, though these changes were not statistically significant (*p* = 0.35) [76].

#### 3.2.3. Effect of Nutraceuticals on Quality of Life

Thirteen RCTs reported the effect of probiotic supplementation on quality of life in patients with AD. Several trials showed significant between-group differences (Table 1). Gerasimov et al. reported a significant reduction in IDQOL scores in the intervention group (Δ = −3.7 ± 3.3) compared to placebo (Δ = −2.3 ± 1.6, *p* = 0.013) [83]. Wang et al. reported reductions in CDLQI with *Lactobacillus paracasei* (Δ = −4.30 ± 8.28, *p* = 0.03) and *Bifidobacterium lactis* (Δ = −4.25 ± 9.80) [74]. Similarly, Carucci et al. [38] found significant improvements in IDQOL (*p* < 0.05), and Wang et al. (2015) [74] observed a reduction in CDLQI with 3-month supplementation of *Lactobacillus paracasei* (Δ = −4.30 ± 8.28) and *Lactobacillus fermentum* (LF) (Δ = −4.25 ± 9.80) with a *p*-value of 0.03. This statistically significant difference between the intervention and placebo groups for these reductions suggests both strains effectively improved QoL [38,74]. Other studies showed mixed results. For example, studies such as Sharma et al. and Grüber et al. showed improvements in both groups but differences between intervention and placebo were not significant [75,85]. Fölster-Holst et al. reported no change in QoL scores [46]. Drago et al. and Yoshida et al. reported significant reductions in DLQI (Δ = −3.86 ± 1.81, *p* = 0.04) and QoL (Δ =−9.6 ± 25.5, *p* = 0.019), respectively [45,50].

#### 3.2.4. Effect of Nutraceuticals on Microbiome Composition

Only 3 RCTs investigated changes in microbiome composition (Table 1). Van der Aa et al. used 16S sequencing to observe that a synbiotic containing *Bifidobacterium breve* M-16V and a galactooligiosaccharides (GOS)/fructooligiosaccharides (FOS) mixture significantly increased *Bifidobacterium* levels (54.7% vs. 30.1%, *p* < 0.001) while reducing potentially inflammatory groups such as *Clostridium lituseburense/C. histolyticum* (0.5% vs. 1.8%, *p* = 0.02) and *Eubacterium rectale/C. coccoides* (7.5% vs. 38.1%, *p* < 0.001) [58]. Carucci et al. reported that supplementation with *Lacticaseibacillus rhamnosus* GG (LGG) significantly increased beneficial genera like *Akkermansia* and *Ruminococcus* while reducing pro-inflammatory families such as *Porphyromonadaceae*, *Enterobacteriaceae*, and *Haemophilus* (*p* < 0.05), similarly, assessed by 16S sequencing [38]. Liu et al., 2025 was the only study to use shotgun sequencing and it showed *that Megamonas funiformis* and *Megamonas hypermegale* increased significantly after FMT treatment compared to the placebo treatment [56].

### 3.3. Psoriasis

#### 3.3.1. Effect of Nutraceuticals on Disease Severity Index

Five RCTs explored the effects of using pro/synbiotics on the PASI scores of psoriatic patients (Table 2). Of these, 3 trials established statistical significance in PASI score improvement. Akbarzadeh et al. showed that in a group of 25 psoriatic patients, 12-week supplementation with Lactocare^®^ probiotic (twice daily) significantly reduced PASI scores (Δ = −3.14 ± 0.67 from baseline compared to Δ = −0.28 ± 0.57 in the placebo group) (*p* = 0.001) [40]. In another trial, Moludi et al. reported a significant PASI reduction of −5.26 ± 3.75 after 8-week supplementation with probiotics in the intervention group, compared to an increase of 0.48 ± 1.37 in the placebo group (*p* = 0.049) [89]. Also, Drouin et al. showed a small but significant reduction (−1.2 ± 1.8) with no change in placebo (Δ = 0.0 ± 0.6) [90]. In contrast, Gilli et al. reported reductions in PASI scores (Δ = −0.96 ± 5.57) in the intervention group, but this change was not statistically significant compared to the placebo (Δ = 0.75 ± 5.44, *p* = 0.59) [91]. Suriano et al. observed PASI reductions of −1.58 in the intervention group compared to a larger and significant reduction of −1.90 in the placebo group, without significant between-group differences (*p* = 0.62) [55]. Overall, the direction and magnitude of effects across trials show modest but inconsistent PASI improvements, with some studies demonstrating significant reductions while others showed minimal or no benefit.

#### 3.3.2. Effect of Nutraceuticals on Inflammatory/Immunological Markers

Only 1 RCT investigated inflammatory markers in patients with psoriasis (Table 2). Gilli et al. investigated the effects of *Lactobacillus rhamnosus* on IL-17 and IL-23 levels. In the intervention group, IL-17 levels increased from 16.97 ± 17.776 pg/mL to 21.15 ± 17.893 pg/mL, while the placebo group showed a decrease from 44.46 ± 59.703 pg/mL to 20.05 ± 21.15 pg/mL [91]. However, neither change was statistically significant. For IL-23, levels in the intervention group increased from 21.5 ± 22.802 pg/mL to 30.49 ± 25.315 pg/mL, whereas the placebo group had a minimal increase from 45.17 ± 51.909 pg/mL to 47.9 ± 88.492 pg/mL. These changes were also not statistically significant. Taken together, cytokine changes were small, variable, and inconsistent in direction, indicating no meaningful or reliable immunomodulatory effect on IL-17 or IL-23.

#### 3.3.3. Effect of Nutraceuticals on Quality of Life

Five RCTs investigated the effect of pro/synbiotics supplementation on the quality of life of patients with psoriasis (Table 2). Of these, 3 trials reported significant improvements in the intervention groups compared to placebo. Akbarzadeh et al. showed that 12-week supplementation with Lactocare^®^ probiotics combined with topical hydrocortisone significantly reduced DLQI scores (Δ = −5.32 ± 1.10, *p* < 0.05) compared to minimal placebo improvements (Δ = 0.89 ± 1.30, *p* > 0.05) with significant in-between group difference of *p* = 0.017 [40]. Similarly, Moludi et al., using a multi-strain probiotic for 8 weeks, reported a significant reduction (Δ = −9.50 ± 4.1, *p* = 0.001) compared to no change in placebo (Δ = −0.12 ± 0.6, *p* = 0.998), with a *p*-value of 0.045 for in-between group change post intervention from baseline [89]. Gilli et al. noted small reductions in both groups (Δ = −1.83 ± 11.65, intervention; Δ = −2.12 ± 11.92, placebo), with a significant between-group difference (*p* = 0.035) despite minimal within-group changes [91]. In contrast, Drouin et al. showed no significant improvements in the intervention group (Δ = 0.9 ± 2.5) and worse outcomes in placebo (Δ = 2.5 ± 3.8, *p* < 0.05) [90]. Suriano et al. reported greater placebo improvement (Δ = −3.33 ± 8.68, *p* = 0.031) compared to minor change in the intervention group (Δ = 0.05 ± 5.02, *p* = 0.873, *p* = 0.107) [55]. Across studies, improvements in DLQI were observed in several trials but varied widely in magnitude, reflecting inconsistent but occasionally notable quality-of-life benefits.

### 3.4. Acne Vulgaris

#### 3.4.1. Effect of Nutraceuticals on Disease Severity Index

Five studies observed the effects of probiotics on total lesion count in patients with acne vulgaris (Table 3). All five trials reported significant changes compared to placebo. Kim et al. showed that, in a cohort of 28 patients, a 12-week supplementation with a probiotic significantly reduced TLC from 72.93 ± 10.11 to 33.57 ± 4.77 (Δ = −53.97 ± 10.04), a change that was highly significant compared to placebo (Δ = −4.29 ± 12.58, *p* = 0.002) [18]. Draelos et al. [92] reported a significant reduction in acne severity by week 12 (IGA 1.47 vs. 1.98, *p* = 0.02), with marked decreases in inflammatory lesions (56.2%) and non-inflammatory lesions (48.1%), alongside significantly improved blinded investigator skin assessments. In another study, Atefi et al., 2025 [42], 80 patients with moderate acne were randomized into two groups: doxycycline alone (control) or doxycycline plus a daily probiotic (intervention). All patients used the same topical regimen [42,92]. While both groups improved on the Global Acne Grading System (GAGS), the doxycycline-plus-probiotics group demonstrated significantly superior outcomes in several facial regions, including the forehead (*p* = 0.018), chin (*p* = 0.021), and nose (*p* = 0.021). No significant differences were observed for the left or right cheeks, back, or chest, with the overall mean GAGS score reduction differing by only 2% between groups. Probiotic treatment significantly reduced overall lesion severity compared to control (*p* < 0.001), and the acne grading method confirmed a markedly better treatment response in the intervention group (*p* < 0.001). Importantly, no side effects were reported with probiotic use. In another trial, Liang et al. showed a reduction in TLC, with a larger decrease of −43.7 ± 2.26 in the intervention group, which received probiotic and isotretinoin and a reduction −41.50 ± 3.61 in the placebo group, which received isotretinoin only, both achieving statistical significance (*p* < 0.001) [41]. Although similar reductions were reported in another study by Eguren et al., the difference between groups did not reach statistical significance (*p* = 0.06) [93].

#### 3.4.2. Effect of Nutraceuticals on Microbiome Composition

Only 1 RCT evaluated microbiome composition in acne vulgaris patients (Table 3). Liang et al. noted that *Lactobacillus plantarum* MH-301 supplementation increased *Lactobacillus* (0.81% vs. 0.09%), *Bifidobacterium* (2.68% vs. 2.09%), *Coprococcus* (7.68% vs. 3.09%), and *Bacteroides* (18.36% vs. 17.31%) levels while reducing *Proteobacteria* (0.84% vs. 1.54%), *Verrucomicrobia* (0.01% vs. 1.37%), and *Faecalibacterium* [41]. The single RCT assessing microbiome composition showed modest shifts toward increased *Lactobacillus*-, *Bifidobacterium*-, and *Verrucomicrobia*-type taxa with reductions in Proteobacteria.

### 3.5. Chronic Urticaria

#### 3.5.1. Effect of Nutraceuticals on Disease Severity Index

Two RCTs investigated the effect of pro/synbiotic supplementation on UAS7 in patients with chronic urticaria (Table 4). Dabaghzadeh et al. showed reductions of −10.6 ± 9.6 in the intervention group and −7.7 ± 11.7 in the control group with statistical significance in-between-group difference (*p* = 0.036) without within-group statistical significance for both groups [94]. However, Atefi et al. reported a change of −18.47 ± 15.63 in the control group compared to −21.00 ± 13.82 in the intervention group but the difference between these changes was not significant (*p* > 0.05) [52]. Across both trials, UAS7 scores showed reductions in intervention groups, but the magnitude of improvement was modest and often comparable to placebo.

#### 3.5.2. Effect of Nutraceuticals on Quality of Life

Two RCTs explored the effect of pro/synbiotic supplementation on the quality of life in urticaria patients (Table 4). Of these, 1 study reported significant improvements compared to the control/placebo, while the other showed no significant differences. Atefi et al. found that supplementation with a LactoCare synbiotic capsule containing multiple probiotic strains and FOS, administered twice daily alongside antihistamines for 8 weeks, resulted in a significant DLQI reduction of −9.43 ± 8.12 in the intervention group versus −7.62 ± 11.29 in the placebo group (*p* = 0.049) [52]. The intervention group’s DLQI decreased from 14.47 ± 6.4 to 5.04 ± 5.00, while the placebo group’s scores decreased from 17.33 ± 6.49 to 9.71 ± 9.24 (*p* < 0.0001), so the within-group difference for each was also significant. Conversely, Dabaghzadeh et al., using a Femilact probiotic capsule containing multiple strains (2 × 10^9^ CFU/day) combined with cetirizine for 8 weeks, showed a DLQI reduction of −3.1 ± 10.82 in the intervention group and −0.9 ± 7.30 in the placebo group, however the between-group difference was not significant (*p* = 0.805) [94]. Quality-of-life outcomes showed variable effects, with one trial demonstrating notable DLQI improvement and the other showing minimal change.

### 3.6. Melasma

#### Effect of Nutraceuticals on Disease Severity Index

One RCT investigated the effect of synbiotic supplementation on the mMASI in patients with melasma (Table 5). Piyavatin et al. showed that in a group of females with melasma, daily supplementation with 1 sachet of synbiotic TS6 (containing 6 probiotic strains and prebiotics) for 12 weeks significantly reduced mMASI (Δ = −0.56 ± 0.06, *p* < 0.001) was observed compared to a slight increase in mMASI (Δ = +0.03 ± 0.12, *p* = 0.592) [95]. The between-group difference was statistically significant (*p* = 0.008). This single trial showed a clear, statistically significant reduction in mMASI with synbiotic supplementation.

## 4. Discussion

Across 5 dermatological conditions, nutraceuticals demonstrated a broadly favorable but condition-dependent impact, with the most consistent improvements observed in atopic dermatitis and melasma, and more variable effects in psoriasis, acne, and chronic urticaria. Many reviews have explored the potential of nutraceuticals in managing skin diseases; however, to our knowledge, this is the most comprehensive review across multiple dermatological conditions [29,33,34,35]. Our results indicate that these interventions have the potential to improve disease severity indices, specifically SCORAD for AD, PASI for psoriasis, and mMASI for melasma under appropriate intervention duration, type of strain, and target populations. While quality-of-life outcomes generally tracked with changes in clinical severity, inflammatory and immunological markers showed inconsistent shifts, showing the need for better alignment between mechanistic endpoints and clinical response. Also, the evidence for their effects on inflammatory and immunological markers, such as IL-17, IL-23, IL-4, IFN-γ, TARC and IgE, remains limited and inconsistent. Additionally, no inflammatory marker or quality of life data were reported for acne vulgaris or melasma.

### 4.1. Gut Microbiome Dysbiosis and Therapeutic Modulation in Atopic Dermatitis

The gut microbiota of AD patients showed reduced diversity and disturbances in SCFA producing taxa compared with healthy individuals. These alterations suggest a shift toward dysregulated immune signaling. Notably, *Faecalibacterium prausnitzii* has been found in elevated levels in AD patients, with a positive correlation to disease severity measured by SCORAD scores [96]. While *F. prausnitzii* is typically associated with anti-inflammatory properties, its disproportionate abundance in AD may reflect a compensatory response or a context-specific role in dysregulated immunity. This dysbiosis provides a biological rationale for testing probiotic strategies aimed at restoring metabolic and immunologic balance

In our review, probiotic interventions, particularly those involving *Lactobacillus* strains, demonstrated modest but variable improvements in AD severity. While some studies showed statistically significant reductions in SCORAD scores, others reported no significant difference from placebo, echoing prior literature that has questioned the consistency of probiotic efficacy in AD [26,50,87]. It is important to show caution regarding some of the studies showing SCORAD improvement. For example, Lin et al., 2015 reported these positive changes following intervention; however, this study was assessed as having an overall high risk of bias due to concerns in randomization process (D1) and selective reporting of outcomes (D5) [53]. Similarly, another study reported improvements in clinical outcomes of AD following intervention [54]. However, due to the risk of bias related to randomization process (D1) and missing outcome data (D3), this study was determined to have high risk of bias. Taken together, these limitations warrant cautious interpretation, but do not preclude discussion of the broader body of evidence. Further, some study interpretation was limited by sample size. Another had a sample size of 12 in the intervention group and 10 in the control group, bringing doubt on the interpretation of these results [61]. Regardless, strain-specific differences were apparent: *L. salivarius*, *L. fermentum*, *L. sakei*, and *L. plantarum* were generally associated with symptomatic relief, whereas *L. paracasei* yielded inconclusive outcomes. Mechanistically, these mixed results may be partially explained by differences in probiotic ability to correct underlying metabolic deficiencies. Children with AD have been shown to harbor gut microbiota with impaired SCFA synthesis capacity [97,98,99], which may blunt the effectiveness of certain strains. Supporting this, butyrate supplementation in a murine model of AD-like inflammation improved barrier integrity, reduced mast cell infiltration, and decreased sensitization through enhanced keratinocyte turnover and increased structural lipid production [100]. These findings are consistent with our review’s observation that SCFA-producing probiotics are associated with improved clinical outcomes in AD.

**Figure 3:**
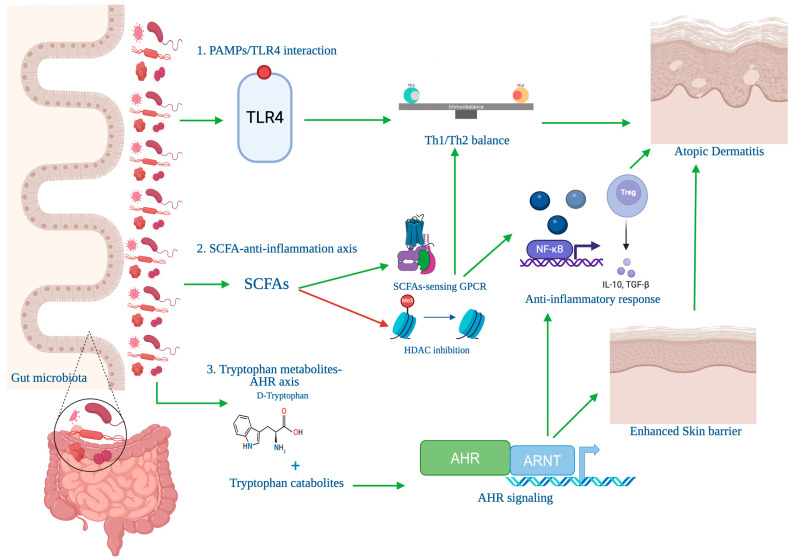
A schematic figure showcasing the mechanisms by which the gut microbiota regulates AD. The figure illustrates 3 major mechanisms: (1) SCFAs-mediated anti-inflammatory axis, where gut-derived SCFAs such as acetate, propionate and butyrate modulate inflammation through GPCR signaling and HDAC inhibition leading to an improved Th1/Th2 balance, activation of NF-κB gene expression and Treg (Regulatory T Cell) cell activation that produce anti-inflammatory cytokine production (IL-10, TGF-β). (2) tryptophan metabolism-AHR axis, where tryptophan metabolites activate the AHR signaling, which also improves Th1/Th2 balance, lead to activation of NF-κB gene expression and T-reg cell and enhanced skin barrier. (3) PAMP/TLR4 interactions, where Pathogen PAMPs from gut microbes engage TLR4 signaling, further impacting Th1/Th2 balance. These pathways collectively regulate Th1/Th2 cytokine balance, reduce skin inflammation, and enhance barrier integrity, providing insights into the gut-skin axis and gut microbiota role in AD management. This figure was designed and created by the authors using Biorender.com. Adapted from [17].

The therapeutic effects of probiotics in AD are supported by multiple mechanistic pathways documented in the literature (Figure 3). In animal studies, SCFAs signal through G-protein-coupled receptors (GPCRs) and inhibit histone deacetylases (HDACs), which modulate inflammatory gene expression and promote Th1/Th2 immune balance. In parallel, tryptophan metabolites produced by gut microbes activate the aryl hydrocarbon receptor (AHR), which has been shown to enhance skin barrier function and reduce inflammation in mouse models. Additionally, microbial components such as pathogen-associated molecular patterns (PAMPs) engage TLRs on host immune cells, further influencing cytokine profiles and attenuating IgE-mediated allergic responses. Together, these immune, metabolic, and epithelial signaling pathways explain how probiotic supplementation may restore immune homeostasis and strengthen epithelial defenses.

Although probiotics have demonstrated potential in managing and preventing AD, the role of prebiotics remains less clear. Although some studies have shown reductions in AD incidence, the World Allergy Organization (WAO) advises caution surrounding their long-term efficacy [17]. In our review, only two studies explored the effects of prebiotics on SCORAD in AD: kestose significantly reduced SCORAD scores, while GOS had no significant impact [60,65]. This disparity may be attributed to differences in fermentation profiles and microbial selectivity. In animal studies, kestose has been shown to selectively promote the growth of *Bifidobacterium* species with enhanced SCFA-producing capacity, particularly butyrate, whereas GOS may favor broader microbial populations without the same functional specificity.

### 4.2. Gut Microbiome Dysbiosis and Therapeutic Modulation in Psoriasis

Psoriasis is linked to distinct gut microbial imbalances, including reductions in *Faecalibacterium* and *Akkermansia*, and expansion of *Ruminococcus* and *Enterobacteriaceae*. Unlike in atopic dermatitis, where SCFA-mediated Treg promotion is theorized to dominate mechanistic models, in psoriasis the pathology is more closely tied to Th17/IL-23 axis activation, intestinal permeability, and microbial metabolite dysregulation.

Synbiotic interventions such as Lactocare^®^ likely provide benefit not only via SCFA production, but also through restoration of trace mineral absorption (e.g., zinc and magnesium), which support keratinocyte differentiation and NF-κB inhibition. Additionally, microbial modulation of bile acid metabolism may influence systemic inflammation by affecting farnesoid X receptor (FXR) signaling, which has been linked to increased gut permeability and elevated levels of circulating pro-inflammatory cytokines in psoriasis models. Moludi et al.’s multi-strain probiotic demonstrated effects on systemic inflammation through reductions in lipopolysaccharide (LPS) and IL-1β, pointing to barrier repair via modulation of tight junction proteins (e.g., claudin-1) and mucin secretion, mechanisms distinct from GALT-centered T-reg enhancement. These effects may be contributed to by bacterial exopolysaccharides that reduce TLR4 pathway activation and modulate dendritic cell tolerance thresholds [89]. Further, Gilli et al.’s use of single-strain *L. rhamnosus* showed limited effect, likely due to insufficient modulation of microbial-derived immunomodulatory metabolites like indole derivatives (e.g., indole-3-aldehyde), which interact with the AhR. Animal studies suggest that probiotics may modulate inflammatory pathways in psoriasis, particularly the IL-23/Th17 axis. Interestingly, one study reported no significant improvement in PASI scores; however, the study had high risk of bias due to concerns of missing outcome data (D3) and selective reporting of outcomes (D5), which limits its generalizability and allows for current theoretical mechanisms to hold weight [89].

Overall, gut-directed therapy in psoriasis appears to act barrier reinforcement and inhibition of the IL-23/Th17 axis, with comparatively less emphasis on the potential Treg-mediated tolerance or tryptophan metabolism, distinguishing its mechanisms from those seen in AD or chronic urticaria.

### 4.3. Gut Microbiome Dysbiosis and Therapeutic Modulation in Acne Vulgaris

Acne vulgaris represents a more important, multifactorial interaction between metabolism, hormones, and inflammation. Gut dysbiosis can exacerbate intestinal permeability and trigger systemic mTOR signaling, promoting perifollicular inflammation [18]. While some probiotics may reduce oxidative stress and pro-inflammatory cytokines, their influence on hormonal drivers like androgens is minimal. As such, their therapeutic impact is likely limited or conditionally effective.

Studies have shown that in acne vulgaris patients, some observed microbial changes include reduced gut microbiota diversity, increased Bacteroidetes: Firmicutes ratio, elevated *Proteobacteria* and decreased *Actinobacteria* and reduced levels of beneficial, anti-inflammatory genera such as *Lactobacillus*, *Bifidobacterium*, *Butyricicoccus*, *Coprobacillus*, and *Allobaculum* [101]. This microbial shift may elevate systemic inflammation through increased intestinal permeability and elevated circulating endotoxins such as LPS, which can stimulate sebaceous glands and contribute to lesion formation.

Several RCTs demonstrate the clinical benefit of probiotics as adjuncts to conventional acne therapy. Eguren et al. (2024) found that a combination of *Lacticaseibacillus rhamnosus* CECT 30031 and arabinogalactooligosaccharides significantly improved the Acne Global Severity Scale (AGSS) by reducing both inflammatory and non-inflammatory lesions, potentially through restoration of gut barrier function and reduction in systemic LPS [93]. Liang et al. (2024) reported that *Lactobacillus plantarum* MH-301 supplementation alongside isotretinoin improved skin outcomes and partially corrected microbial imbalance by increasing the abundance of beneficial bacteria [41].

Mechanistically, SCFAs like butyrate may reduce sebocyte activity, and inflammation, leading to improved acne outcomes. Furthermore, animal studies show that *Lactobacillus plantarum* reduces expression of TNF-α and IL-8 in skin models, suggesting systemic anti-inflammatory effects that extend from the gut to the dermis [18]. The gut–brain-skin axis may also be relevant in acne; certain probiotics enhance GABA production, reducing stress-related flares by modulating neuroendocrine signaling. Another study highlighted the effect of probiotics on the skin through animal models. Mice were put under stressed conditions, then received oral *Lactobacillus reuteri* with results showing reduced rates of perifollicular inflammation compared to control [102]. Decrease in major histocompatibility cell (MHC) class II expression surrounding hair follicles was also observed. This is crucial since perifollicular inflammation is one of the early steps of acne development [103].

### 4.4. Gut Microbiome Dysbiosis and Therapeutic Modulation in Urticaria

Chronic urticaria is increasingly recognized as an immune-mediated disorder involving both mast cell activation and systemic inflammation. Gut dysbiosis in chronic urticaria has been characterized by decreased levels of *Bifidobacterium* and *Lactobacillus*, along with reduced SCFA-producing taxa [8]. This disruption may impair gut barrier integrity and enhance systemic allergen exposure.

Two RCTs evaluated the role of probiotics in chronic urticaria. Dabaghzadeh et al. (2023) [94] reported a significant decrease in UAS7 scores following 8 weeks of supplementation with *Lactobacillus acidophilus*, *B. bifidum*, and *Streptococcus thermophilus*. Atefi et al. (2022) [52] showed that LactoCare^®^ synbiotic therapy (which includes *L. casei*, *L. rhamnosus*, and inulin) improved quality of life and symptom severity. However, the study was assessed as having an overall high risk of bias due to concerns in the randomization process (D1) and selective reporting of outcomes (D5), which limits confidence in the reported outcomes. Despite these limitations, these outcomes are biologically plausible and suggest that microbial modulation may reduce histamine release and mast cell sensitization through Treg expansion and reduction in circulating IL-4. The benefit of synbiotics in urticaria may lie in their capacity to restore gut immune tolerance. SCFAs such as acetate and propionate theoretically promote IL-10-producing Tregs and reduce Th2 polarization. Furthermore, microbial modulation of the serotonin pathway may attenuate neurogenic inflammation, which has been implicated in chronic urticaria pathogenesis. In murine models, SCFA supplementation was shown to reduce mast cell degranulation and histamine release, suggesting a functional gut-skin immunological axis.

### 4.5. Gut Microbiome Dysbiosis and Therapeutic Modulation in Melasma

Melasma, a hyperpigmentary disorder often exacerbated by UV exposure and hormonal fluctuations, has emerging links to the gut microbiota. Although limited, available evidence suggests dysregulation in bile acid metabolism and SCFA profiles may indirectly influence melanogenesis by affecting systemic oxidative stress and estrogen metabolism.

In a double-blind RCT, Piyavatin et al. (2021) [95] demonstrated that a synbiotic containing six probiotic strains (*L. bulgaricus*, *L. acidophilus*, *B. longum*, among others) with FOS significantly reduced mMASI scores in patients with melasma. This improvement was accompanied by reductions in skin erythema and melanin indices. Mechanistically, synbiotic-driven changes in the gut microbiota may enhance FXR signaling and reduce systemic oxidative load, which in turn downregulates tyrosinase activity and melanocyte proliferation.

Additionally, probiotics may reduce local skin pigmentation through tryptophan metabolism and AhR signaling, seen in animal models. Butyrate-producing microbes also modulate melanocyte activity via epigenetic mechanisms such as histone acetylation at pigment-associated gene promoters. This suggests a potential gut-derived checkpoint in pigmentation pathways. Though no direct animal studies were cited in this study, prior murine data suggest that modulation of gut microbes can attenuate UV-induced pigmentation through suppression of pro-melanogenic cytokines such as IL-1β and TNF-α.

### 4.6. Overlap and Divergence

Despite distinct clinical manifestations, acne, melasma, urticaria, atopic AD, and psoriasis share similar features of gut dysbiosis, like reduced microbial diversity, impaired barrier integrity, and diminished SCFA production. This disruption nurtures systemic inflammation and weakens immune regulation, particularly through reduced Treg differentiation via SCFA-GPR signaling pathways [8,89].

Each condition presents distinct downstream effects based on its underlying pathophysiology. In psoriasis, for instance, the overactivation of the IL-23/Th17 pathway makes it particularly responsive to nutraceuticals aimed at dampening Th17 responses and strengthening the gut barrier [89]. AD, which is characterized by Th2 dominance and IgE-driven inflammation, tends to benefit more from SCFA-producing probiotics that promote Treg activity and reduce allergic inflammation [104]. Chronic urticaria, on the other hand, is primarily driven by mast cell degranulation and histamine release. While SCFAs may help stabilize mast cells and mitigate neurogenic inflammation, the role of nutraceuticals in this condition appears to be more supportive than central [105].

Acne vulgaris has a complex interaction between metabolism and the immune system. Gut dysbiosis can increase intestinal permeability and activate systemic mTOR signaling, which contributes to perifollicular inflammation [18]. While probiotics may offer mild improvement by reducing oxidative stress and inflammatory cytokines, they generally have limited influence on the hormonal imbalances that drive acne. Melasma, by contrast, involves a different mechanism: alterations in the gut microbiota affect bile acid metabolism and estrogen recycling. These changes impact melanogenesis through FXR signaling and modulation of oxidative stress [95].

Overall, while nutraceuticals consistently enhance gut barrier and reduce inflammation, their efficacy varies based on the condition’s underlying mechanism. Conditions that are driven by inflammatory issues, like psoriasis and AD, respond well, whereas metabolically or hormonally influenced diseases like acne and melasma show more modest improvements.

### 4.7. Limitations

This systematic review is subject to limitations that need to be considered. One major issue is that there are different numbers of studies for the various skin diseases examined. For instance, there are 47 studies related to AD but only 5 psoriasis, 5 acne vulgaris, 2 chronic urticaria, 1 study focused on melasma. This disparity may limit the generalizability and comparative strength of the conclusions drawn across conditions. Further, restricting the review to English-language publications may introduce language bias and limit the capture of relevant evidence published in other regions. This exclusion may also reduce the generalizability of findings, as studies using different cultural or geographic populations were not assessed.

In addition, we observed substantial heterogeneity among the included RCTs. Participant populations varied widely in terms of age, ethnicity, medical conditions, and other relevant information. Baseline disease severity was not consistently reported or accounted for, even though it represents a potentially important confounding factor [106]. The studies also varied considerably in their interventions, including differences in probiotic strains, prebiotic and synbiotic formulations, as well as treatment durations. Additionally, the studies reported a wide range of outcomes, from clinical indices such as SCORAD and PASI to various inflammatory biomarkers, complicating efforts to compare results across trials.

Another limitation of this systematic review relates to the influence of placebo effects observed in the included RCTs. Participants in placebo groups often demonstrated notable improvements in disease severity scores, inflammatory biomarkers, and quality of life outcomes, which complicates the interpretation of the interventions’ efficacy. Additionally, long-term follow-up data were scarce, with only four trials assessing outcomes beyond the treatment period, further limiting the understanding of the durability of therapeutic benefits. Reporting on tolerability was also inconsistent, with common side effects such as nausea and constipation either underreported or omitted altogether. Furthermore, this review did not include interventions such as fecal microbiota transplantation (FMT) or postbiotics, due to the limited number of studies available on these modalities in dermatologic populations.

Finally, safety and tolerability data were inconsistently reported across the included trials. Most studies that documented adverse events reported mild and transient gastrointestinal symptoms, with no serious adverse events attributed to the interventions. However, the absence of systematic safety reporting in many trials limits firm conclusions about tolerability. Future studies should incorporate standardized and comprehensive adverse event monitoring to better inform clinical use.

### 4.8. Future Perspectives

To better understand how the gut microbiota influences inflammatory skin conditions, further mechanistic studies are needed. Future research should focus on microbial metabolites such as short-chain fatty acids and tryptophan derivatives, along with immune pathways including the Th17/IL-23 and Treg axes. Clarifying the causal relationship between microbial dysbiosis and cutaneous inflammation can be very helpful in developing targeted therapeutic strategies.

We observed methodological inconsistencies across RCTs, which limit the comparability of findings between studies. These include variations in patient selection, outcome measures, treatment duration, and sequencing techniques. While initiatives such as the Human Microbiome Project have advanced the field [107], there remains a need to address skin-specific confounders, such as unreported use of topical products or environmental exposures, that may influence results and hinder broader conclusions.

Nutraceuticals may require regional and cultural adaptation to optimize their therapeutic efficacy. Dietary habits, lifestyle factors, and environmental exposures differ significantly across populations and can influence the composition and function of the gut microbiota. For instance, the high consumption of fermented foods in East Asian populations compared to the more processed, high-fat diets common in Western countries may result in distinct baseline microbial profiles. Consequently, the effectiveness of specific probiotics, prebiotics, or synbiotics may vary across regions and ethnic groups. This is exemplified in the context of acne vulgaris, where two randomized controlled trials using L. plantarum MH-301 reported beneficial effects in improving acne symptoms. However, both studies were conducted in China with relatively homogenous participant populations. This raises an important question: Can the same microbial strain exert similar therapeutic effects in ethnically diverse populations with different microbiome compositions and environmental backgrounds?

There is still a strong need for large, well-designed randomized controlled trials to determine the long-term safety and actual effectiveness of nutraceuticals. This includes not only established strategies like probiotics and prebiotics but also newer approaches such as synbiotics, postbiotics, and FMT for chronic inflammatory skin diseases. Future studies should work to incorporate longer follow-up periods and consistent methods for monitoring and keeping up with adverse events to ensure reliable and clinically meaningful results. Tailoring interventions according to patient-specific microbiome and immune profiles could better enhance treatment outcomes and minimize unnecessary medication exposure.

## 5. Conclusions

This systematic review examined 60 RCTs evaluating the use of nutraceuticals, probiotics, prebiotics, and synbiotics, on five skin diseases: AD, psoriasis, acne vulgaris, chronic urticaria, and melasma. These interventions in certain diseases appear to reduce symptoms’ severity, improve overall quality of life, and possibly reduce systemic inflammation. However, these findings should be interpreted cautiously given the generally small sample sizes, variable methodological quality, short intervention durations, and heterogeneity in outcome reporting. Some diseases have minimal literature on them, making it difficult to draw conclusions. Evidence gaps differ across conditions: AD studies require clearer mechanistic endpoints and longer-term follow-up; psoriasis trials need larger samples and standardized inflammatory markers; acne research lacks robust trials integrating microbiome and metabolic outcomes; chronic urticaria studies require clearer definitions of clinical response and better placebo-controlled designs; and melasma evidence remains limited to single small RCTs requiring replication. There is also a need for deeper mechanistic insights to clarify how different probiotic, prebiotic, and synbiotic formulations exert their effects. Such understanding would guide the selection of the most effective combinations for each specific skin condition. Overall, the literature presented mixed results across skin diseases, showing the need for well-designed randomized controlled trials to determine optimal strains, dosages, and durations for specific diseases. Future studies should use rigorously designed, strain-specific RCTs with adequate sample sizes, longer follow-ups, and standardized outcomes to improve comparability. Trials that include mechanistic endpoints and compare different probiotic, prebiotic, and synbiotic formulations will help clarify optimal strategies for each skin condition.

## Figures and Tables

**Table 1:** Study characteristics and outcomes of nutraceutical interventions on AD patients including author, country, intervention (strain/dose), type, duration, route, control/placebo, participant demographics, and primary/secondary outcomes.

**Author**	Country	Intervention + Dose	Type of Intervention	Intervention Duration	Route of Intervention	Control/Placebo	Intervention Patient Demographics (n, M/F, Mean Age (SD), BMI)	Controls Patient Demographics (n, M/F, Mean Age (SD), BMI)	Primary Outcome	Secondary Outcome
Shafiei 2011 [57]	Iran	10 mg mixture of 1 × 10^9^ CFU/ of 7 strain probiotics plus prebiotic (990 mg fructooligosaccharides)	Synbiotic	2 months	Oral	Daily one sachet containing 1000 mg sucrose	18, 12/6, 14.7 (6) months	18, 12/6, 15.4 (8.4) months	SCORAD	Total IgE level and Eosinophil Count
Va Der Aa 2010 [58]	The Netherlands	*B. breve* M-16V, 1.3 × 10^9^ CFU/100 mL and a mixture of 90% scGOS and 10% lcFOS, 0.8 g/100 mL	Synbiotic	12 weeks	Oral	Infants received the same formula without synbiotics	46, 31/15, 5 (1.4) months	44, 28/16, 4.8 (1.5) months	SCORAD	Topical Corticosteroid Usage, Total IgE, Eosinophilic granulocytes, Microbiota Composition, and Stool Characteristic, gastrointestinal symptoms, and adverse events
Wu 2017 [59]	Taiwan	*Lactobacillus rhamnosus* (MP108), 350 mg/day	Probiotic (*Lactobacillus rhamnosus*)	8 weeks	Oral	Placebo (Maltodextrin capsule)	33 children, 31/15, mean age 1.5 years (SD = 1.1)	33 children, 28/16, mean age 1.8 years (SD = 1.1)	SCORAD	Infant Dermatitis Quality of Life Questionnaire, Dermatitis Family Impact Questionnaire
Boženský 2015 [60]	Czech Republic	Hypoallergenic formula supplemented with galacto-oligosaccharides (0.5 g/100 mL)	Prebiotic (galacto-oligosaccharides)	6 months	Oral	Hypoallergenic formula without prebiotics	52, 31/21, 6–8 weeks (no SD provided), BMI: Not applicable (infants)	51, 36/15, 6–8 weeks (no SD provided), BMI: Not applicable (infants)	SCORAD	Anthropometry, infections, Stool frequency, Vomiting, adverse effects
Cukrowska 2021 [43]	Poland	*Lactobacillus rhamnosus* ŁOCK 0900, *Lactobacillus rhamnosus* ŁOCK 0908, and *Lactobacillus casei* ŁOCK 0918 1 billion CFU/day	Probiotic	3 months	Oral	Maltodextrin (placebo)	66, 37/29, 8.2 ± 6.1 months, BMI: Not applicable	68, 48/29, 8.8 ± 6.6 months, BMI: Not applicable	SCORAD	Levels of total serum IgE and allergen-specific IgE
Carucci 2022 [38]	Italy	*Lacticaseibacillus rhamnosus* GG (LGG) at 1 × 10^10^ CFU/daily	Probiotic	12 weeks	Oral	Placebo capsule (identical in appearance and dosage)	46, 31/15, 18.9 ± 8.6 months, BMI: Not reported	45, 33/12, 16.4 ± 7.4 months, BMI: Not reported	SCORAD	Number of Days Without Rescue Medications, IDQOL and Gut and Skin Microbiome
Nakata 2019 [44]	Japan	*Lactobacillus acidophilus* L-92 (20 mg/day, 2 × 10^10^ cells)	Paraprobiotic	24 weeks	Oral	Placebo with a low dose of 0.2 mg L-92	25, 16/9, 1.7 years (0.9–3.0), BMI: Not reported	20, 12/8, 1.8 years (0.9–2.7), BMI: Not reported	SCORAD	Total IgE level, TARC level
Prakoeswa 2022 [39]	Indonesia	*Lactobacillus plantarum* IS-10506 (2 × 10^10^ CFU/day)	Probiotic	8 weeks	Oral	Placebo (skim milk-Avicel)	15, 4/11, 37.67 ± 15.92 years, BMI: Not reported	15, 5/10, 38.07 ± 12.83 years, BMI: Not reported	SCORAD	Serum IgE Levels and Levels of IL-4, IFN-γ, IL-17, Foxp3
Prakoeswa 2017 [61]	Indonesia	Microencapsulated *Lactobacillus plantarum* IS-10506 (10^10^ CFU/day)	Probiotic	12 weeks	Oral	Placebo (skim milk-Avicel)	12, 5/7, 5.7 ± 4.06 years, BMI: Not reported	10, 7/3, 6.02 ± 4.42 years, BMI: Not reported	SCORAD	Serum IgE Levels and Cytokine Levels
D’Auria 2021 [62]	Italy	8 g of rice flour fermented with heat-killed *Lactobacillus paracasei* CBA L74, daily	Probiotic	12 weeks	Oral	Placebo (rice powder without probiotics)	26, 20/6, 12 months (IQR 6–16 months), BMI: Not reported	27, 14/13, 12 months (IQR 8–23 months), BMI: Not reported	SCORAD	Steroid Usage
Michelotti 2021 [63]	Italy	Mixture of probiotics (*Lactobacillus plantarum* PBS067, *Lactobacillus reuteri* PBS072, and *Lactobacillus rhamnosus* LRH020), 1 × 10^9^ CFU per strain	Probiotic	56 days	Oral	Placebo (corn starch and vegetable magnesium stearate)	40, 7/33, 39 ± 1.8 years, BMI: Not reported, SCORAD (Baseline): 20.9 ± 0.5	40, 5/35, 38 ± 1.4 years, BMI: Not reported, SCORAD (Baseline): 19.7 ± 0.4	SCORAD	Skin Smoothness, TEWL, Skin Moisturization, TNF-α, TARC, TSLP levels
Yamamoto 2016 [64]	Japan	Heat-killed *Lactobacillus acidophilus* L-92 (20.7 mg/day)	Probiotic (heat-killed)	24 weeks	Oral	Placebo (tablet containing maltose, starch, and vegetable oil)	24, not specified, 25.5 years, BMI: Not reported	26, not specified, 27 years, BMI: Not reported	SCORAD	EASI, IGA, Serum LDH
Shibata 2009 [65]	Japan	Kestose (1 g for infants <1 year; 2 g for infants >1 year)	Prebiotic (fructo-oligosaccharide)	12 weeks	Oral	Placebo (maltose)	15, 9/6, 17.0 ± 9.4 months, BMI: Not reported	14, 10/5, 17.4 ± 9.1 months, BMI: Not reported	SCORAD	Fecal Bifidobacteria Count and Serum IgE Levels
Yan 2019 [66]	Taiwan	Heat-treated *Lactobacillus paracasei* GM-080, 1 × 10^10^ CFU/day	Probiotic (heat-treated)	16 weeks	Oral	Placebo (maltodextrin)	62, 44/18, 13.48 ± 7.72 months, BMI: Not reported	61, 37/24, 14.62 ± 7.88 months, BMI: Not reported	SCORAD	TEWL, CCL17/TARC Levels, and IgE Levels
Woo 2010 [67]	Korea	*Lactobacillus sakei* KCTC 10755BP (5 × 10^9^ CFU), twice daily	Probiotic	12 weeks	Oral	Placebo (microcrystalline cellulose)	41, 20/21, 6.3 years, BMI: Not reported	34, 13/21, 5.8 years, BMI: Not reported	SCORAD	CCL17/TARC and CCL27 and Use of Topical Corticosteroids
Feito-Rodriguez 2023 [68]	Spain	Mixture of *Bifidobacterium lactis*, *Bifidobacterium longum*, and *Lactobacillus casei*, 1 × 10^9^ CFU per capsule	Probiotic	12 weeks	Oral	Placebo (maltodextrin powder)	35, 22/13, 8.43 ± 3.28 years, BMI: Not reported	35, 24/11, 8.40 ± 3.77 years, BMI: Not reported	SCORAD	IGA and Use of Topical Corticosteroids
Inoue 2014 [69]	Japan	Heat-killed *Lactobacillus acidophilus* L-92, 20.7 mg/day	Probiotic (heat-killed)	8 weeks	Oral	Placebo (maltose, starch, vegetable oil)	24, 14/10, 29.6 ± 13.8 years, BMI: Not reported	25, 14/11, 29.7 ± 14.5 years, BMI: Not reported	SCORAD	Eosinophil count and TGF-β levels
Chernyshov 2009 [70]	Ukraine	*Lactobacillus rhamnosus* R0011 (95%) and *Lactobacillus helveticus* R0052 (5%), 2 billion CFU per capsule, once daily	Probiotic	30 days	Oral	Placebo (maltodextrin)	30, 18/12, 18.43 ± 11.94 months, BMI: Not reported	28, 17/11, 18.21 ± 10.51 months, BMI: Not reported	SCORAD	Topical Steroid Usage and Specific IgG4 to Cow’s Milk
Ahn 2020 [71]	Korea	*Lactobacillus pentosus* (1.0 × 10^10^ CFU), twice daily	Probiotic	12 weeks	Oral	Placebo (similar in taste, smell, and appearance to the probiotic)	41, 25/16, 4.8 ± 2.3 years	41, 12/29, 5.4 ± 3.0 years, BMI: Not reported	SCORAD	Blood Eosinophil Counts and Total Serum IgE
Yeşilova 2012 [72]	Turkey	Combination of *Bifidobacterium bifidum*, *Lactobacillus acidophilus*, *Lactobacillus casei*, and *Lactobacillus salivarius* (2 × 10^9^ CFU/day)	Probiotic	8 weeks	Oral	Placebo (skim milk powder and dextrose)	20, 12/8, age ranged from 1–13 years, BMI: not reported	20, 11/9, age ranged from 1–13 years, BMI: not reported	SCORAD	Levels of IL-5, IL-6, IFN-γ and Total serum IgE
Isolauri 2000 [73]	Finland	*Bifidobacterium lactis* Bb-12 or *Lactobacillus* GG (3 × 10^8^ CFU/g or 1 × 10^9^ CFU/g), included in extensively hydrolyzed whey formula	Probiotic	2 months	Oral (formula feeding)	Placebo (extensively hydrolyzed formula)	18, 4.6 months, BMI: Not reported	9, age not specified, 4.6 months, BMI: Not reported	SCORAD	EPX in Urine and Serum CD4 levels
Wang 2015 [74]	Taiwan	*Lactobacillus paracasei* (LP) and *Lactobacillus fermentum* (LF), either alone or in combination (2 × 10^9^ CFU/day)	Probiotic	3 months	Oral	Placebo	165, M/F: not provided, 7.8 years (SD: 3.79–8.34), BMI: Not reported	55, M/F: not provided, 8.04 years (SD: 3.97), BMI: Not reported	SCORAD	Quality of Life (FDLQI & CDLQI), Cytokine Levels, IgE Levels, Fecal Microbiota
Sharma 2022 [75]	India	*Bacillus clausii* (Strains O/C, N/R, SIN, and T), 2 billion spores/5 mL, twice daily	Probiotic	8 weeks	Oral (suspension)	Conventional treatment (topical corticosteroids, calcineurin inhibitors, etc.)	57 (49 completed the study), M/F: Not specifically provided, 5.75 ± 3.34 years, BMI: Not reported	57 (54 completed the study), M/F: Not specifically provided, 5.33 ± 4.10 years, BMI: Not reported	SCORAD	IL-17A Levels and Quality of Life (CDLQI and IDQOL)
Rosenfeldt 2003 [76]	Denmark	*Lactobacillus rhamnosus* 19070-2 and *Lactobacillus reuteri* DSM 122460 (10^10^ CFU of each strain twice daily)	Probiotic	6 weeks	Oral (dissolved in water)	Placebo (skimmed milk powder and dextrose)	43, 18/255.2 years (range: 1–13 years), BMI: Not reported	43, 18/25 5.2 years (range: 1–13 years), BMI: Not reported	SCORAD	Serum Eosinophil Cationic Protein (sECP) and levels of IL-2, IL-4, IL-10, IFN-γ
Iemoli 2012 [77]	Italy	*Lactobacillus salivarius LS01* and *Bifidobacterium breve* BR03 (1 × 10^9^ CFU/day for each strain)	Probiotic	12 weeks	Oral	Placebo (maltodextrin)	32, 14/18, 32.44 ± 1.47 years, BMI: Not reported	16, 6/10, 30.91 ± 2.79 years, BMI: Not reported	SCORAD	Dermatology Life Quality Index (DLQI), Plasma LPS levels, CD8/CD38/CD45RO T Lymphocytes
Andrade 2022 [78]	Brazil	Mixture of probiotics (*Lactobacillus rhamnosus*, *Lactobacillus acidophilus*, *Lactobacillus paracasei*, and *Bifidobacterium lactis*), 1 g per day (once a day)	Probiotic	6 months	Oral	Placebo (maltodextrin sachet)	24, 8/16, Mean age (SD): Not provided, Age: 2–6 years: 7 children, 6–12 years: 10 children, Adolescents: 7 children, BMI: Not reported	16, 8/8, Mean age (SD): Not provided, Age: 2–6 years: 4 children, 6–12 years: 9 children, Adolescents: 3 children, BMI: Not reported	SCORAD	Serum IgE Levels, Skin Prick Test (SPT), Cytokine Levels and Topical Immunosuppressant Use
Prescott 2005 [79]	Australia	*Lactobacillus fermentum* VRI 003 PCC (1 × 10^9^ CFU) twice daily	Probiotic	8 weeks	Oral	Placebo (maltodextrin)	26, 13/13, 11.6 (4.3) months, BMI: Not reported	27, 16/11, 10.4 (3.32) months, BMI: Not reported	SCORAD	IFN-γ levels, TNF-α levels, IL-13 levels
Kirjavainen 2003 [80]	Finland	Viable LGG Group: *Lactobacillus rhamnosus* GG (1 × 10^9^ CFU/g, resulting in a daily intake of 3 × 10^10^ CFU/kg body weight) and Heat-inactivated LGG Group: Heat-inactivated *Lactobacillus rhamnosus* GG	Probiotic	7.5 weeks	Oral (mixed with extensively hydrolyzed whey formula)	Extensively hydrolyzed whey formula (placebo group)	Viable LGG Group: 14, M/F: Not specified, Mean age (IQR): 5.5 months (3.5–6.8 months) and Heat-inactivated LGG Group: 13, M/F: Not specified, Mean age (IQR): 5.5 months	8, M/F: Not specified, Mean age (IQR): 5.5 months	SCORAD	Adverse Gastrointestinal Symptoms and Fecal Microbiota
Jeong 2022 [81]	South Korea	*Lactobacillus rhamnosus* IDCC 3201 Tyndallizate (RHT3201), 1 × 10^10^ CFU/day	Probiotic (heat-killed)	12 weeks	Oral (administered in sachet form)	Placebo (matching sachet without probiotic)	33, 18/15, mean age: 5.67 ± 3.30 years	33, 12/21, mean age: 5.33 ± 2.53 years	SCORAD	Eosinophil Cationic Protein (ECP), Interleukin-31 (IL-31) and Eosinophil Count
Navarro-Lopez 2018 [82]	Spain	Mixture of *Bifidobacterium lactis* CECT 8145, *Bifidobacterium longum* CECT 7347, and *Lactobacillus casei* CECT 9104, 1 × 10^9^ CFU/day	Probiotic	12 weeks	Oral (capsules)	Placebo (maltodextrin capsules)	26, 13/13, 9.35 ± 3.58 years, BMI: Not reported	24, 11/13, 8.96 ± 3.94 years, BMI: Not reported	SCORAD	Topical Steroid Use and Eosinophil Count
Gerasimov 2010 [83]	Ukraine	A mixture of Lactobacillus acidophilus DDS-1, *Bifidobacterium lactis* UABLA-12, and fructo-oligosaccharide, administered at 5 billion CFU twice daily	Probiotic	8 weeks	Oral (powder form reconstituted in water, juice, or baby food)	Identical rice maltodextrin powder without active probiotics	43, 28/1525.6 ± 7.7 months, BMI: Not reported	46, 28/19 24.1 ± 6.3 months, BMI: Not reported	SCORAD	Infant Dermatitis Quality of Life (IDQOL) and Dermatitis Family Impact (DFI)
Viljanen 2005 [84]	Finland	*Lactobacillus* GG (LGG) 5 × 10^9^ CFU	Probiotic	4 weeks	Oral (capsules mixed with food twice daily)	Placebo containing inert matrix material, microcrystalline cellulose	80, M/F: Not provided, Age: Mean 6.4 months, BMI: Not reported	76, M/F: Not provided, Age: Mean 6.8 months, BMI: Not reported	SCORAD	
Grüber 2007 [85]	Germany	*Lactobacillus rhamnosus* GG (LGG) 5 × 10^9^ CFU, administered twice daily	Probiotic	12 weeks	Oral (capsules mixed with milk or water)	Placebo capsules without LGG	54, 7.7 months, 39/15	48, 7.0 months, 30/18	Symptom Load Score (SLS)	SCORAD subscores, Use of Rescue Medication, IgE Levels, Quality of Life
Gore 2012 [26]	United Kingdom	Daily administration of a freeze-dried sachet containing either *Lactobacillus paracasei* CNCM I-2116 or *Bifidobacterium lactis* CNCM I-3446 at 10^10^ CFU per dose	Probiotic	12 weeks	Oral (sachet mixed with liquid)	Placebo sachet containing maltodextrin with no active probiotic	*Lactobacillus paracasei* Group: 45 infants, average age 19 weeks, 28 males/17 females and *Bifidobacterium lactis* Group: 45 infants, average age 20.5 weeks, 24 males/21 females	47 infants, average age 20 weeks, 28 males/19 females	SCORAD	IDQoL, allergic sensitization rates, stool presence of probiotics, urinary eosinophilic protein X (U-EPX), gastrointestinal permeability, or respiratory/allergy symptoms
Han 2012 [86]	South Korea	*Lactobacillus plantarum* CJLP133 at a dosage of 5 × 10^9^ CFU per dose, taken twice daily	Probiotic	12 weeks	Oral (sachet form)	Placebo with identical appearance and taste	58 participants, average age 4.6 years (SD = 3.3), 34 males/24 females	60 participants, average age 5.1 years (SD = 3.3), 35 males/25 females	SCORAD	Total Eosinophil Count, Total IgE Levels, IFN-γ and IL-4 levels, Topical Corticosteroid use, Presence of Probiotic Strain in Fecal Samples
Drago 2011 [45]	Italy	Twice daily dose of *Lactobacillus salivarius* LS01 (DSM 22775) at 1 × 10^9^ CFU per gram, administered in sachet form	Probiotic	16 weeks	Oral (sachet dissolved in water or cold liquid)	Placebo sachets containing only maltodextrin	19 participants, average age 32.07 years (SD = 1.79), 10 males/9 females	19 participants, average age 28.86 years (SD = 2.15), 8 males/11 females	SCORAD	DLQI, Total Serum IgE Levels, Cytokine Production in PBMCs (IL-4 and IFN-γ), Th1/Th2 Cytokine Ratio (IFN-γ + IL-12/IL-4 + IL-5), Fecal Microflora Changes
Lin 2015 [53]	China	*Bifidobacterium bifidum* triple viable capsules, taken at a dosage of one capsule three times daily	Probiotic	4 weeks	Oral (capsules)	No placebo capsules were administered to the control group	20 infants, average age 11.45 months (SD = 7.87), 9 males/11 females	20 infants, average age 12.26 months (SD = 8.31), 12 males/8 females	SCORAD	*Bifidobacterium bifidum* Levels in Stool
Farid 2011 [54]	Iran	Synbiotic mixture containing (*Lactobacillus casei*, *Lactobacillus rhamnosus*, *Streptococcus thermophilus*, *Bifidobacterium breve*, *Lactobacillus acidophilus*, *Bifidobacterium infantis*, and *Lactobacillus bulgaricus*) with Fructooligosaccharide (FOS) as a prebiotic. Administered as 1 billion CFU in freeze-dried powder form, taken twice daily	Synbiotic	8 weeks	Oral (powder reconstituted with water or breast milk)	Placebo powder	19 children, mean age 28.68 months (SD = 40.86), 11 males/8 females	21 children, mean age 22.76 months (SD = 34.03), 14 males/7 females	SCORAD	IL-4 and IFN-γ, Skin Prick Test Sensitization, Adverse Events
Fölster-Holst 2006 [46]	Germany	*Lactobacillus rhamnosus* strain GG, administered at 5 × 10^9^ CFU twice daily, mixed with milk or water depending on the age of the infant	Probiotic	8 weeks	Oral (capsules)	Placebo capsules containing microcrystalline cellulose	26 infants, median age 16.5 months (range 1–53), gender ratio F/M = 9/17	27 infants, median age 21 months (range 5–55), gender ratio F/M = 10/17	SCORAD	VAS Scores, Corticosteroid and Antihistamine Use, Quality of Life and Immunological Markers (ECP, IgE, sCD30)
Brouwer 2006 [47]	Netherlands	Nutrilon Pepti formula with *Lactobacillus rhamnosus* at 5 × 10^9^ CFU/100 mL and *Lactobacillus* GG at 5 × 10^9^ CFU/100 mL	Probiotic	3 months	Oral (hydrolyzed infant formula)	Nutrilon Pepti without probiotics	(*Lactobacillus rhamnosus*): 17 infants, M/F ratio not specified, mean age under 5 months (SD not specified), BMI not reported. *Lactobacillus* GG): 16 infants, M/F not specified, mean age under 5 months, BMI not reported	17 infants, M/F ratio not specified, mean age under 5 months, BMI not reported	SCORAD	Total IgE levels, specific food IgEs, skin prick tests, blood eosinophils, urinary eosinophil protein X (EPX), and cytokine production (IL-4, IL-5, IFN-gamma)
Rather 2021 [48]	South Korea	Live Cells Group: Freeze-dried *Lactobacillus sakei* proBio65 live cells at a dose of 1 × 10^10^ and Dead Cells Group: Freeze-dried, heat-killed *Lactobacillus sakei* proBio65 dead cells at the same dose as the live cells group	Probiotic (Live and Dead Cells)	12 weeks	Oral (sachets)	Microcrystalline cellulose with no active probiotic ingredient	Live Cells Group: 16 (9 males, 7 females), mean age 9.19 ± 4.97 years and Dead Cells Group: 22 (9 males, 13 females), mean age 9.18 ± 4.53 years	20 (9 males, 11 females), mean age 10.10 ± 4.49 years	SCORAD	Intensity score, SCORAD subjective scores, Investigator’s Global Assessment (IGA), moisture and sebum levels, and eosinophils, IgE, ECP, CCL17, CCL27 levels
Nermes 2011 [49]	Finland	Probiotic formula containing *Lactobacillus rhamnosus* GG (LGG) at a dose of 3.4 × 10^9^ CFU daily, mixed with an extensively hydrolyzed casein formula	Probiotic	3 months	Oral (administered with formula)	Identical casein hydrolysate formula without LGG	19 infants, 10/9, mean age 6.4 months (range 2.5–13)	20 infants, 12/8, mean age 7.1 months (range 2.2–12.5)	SCORAD	IgA, IgG, IgM secreting cells, memory B cells (CD19+CD27+ counts), gut and skin microbiota composition, skin prick test (SPT) for allergen sensitivity, and infection rates
Weston 2005 [87]	Australia	*Lactobacillus fermentum* VRI-033 PCC at a dose of 1 × 10^9^ CFU, administered twice daily	Probiotic	8 weeks	Oral (sachets)	Identical maltodextrin powder without active probiotics, given twice daily	28 children, 14/14, mean age 11.5 months (SD = 4.2),	28 children, 16/12 mean age 10.3 months (SD = 3.23)	SCORAD	DFIQ scores, topical corticosteroid usage, parental perception of AD improvement, and lower respiratory tract infection rates
Yoshida 2010 [50]	Japan	*B. breve* (1.0 × 10^10^ CFU)	Probiotic	8 weeks	Oral	Placebo is Test sample B, capsules in which the Test sample A (capsules filled with a lyophilized powder of live *B. breve* strain YY) constituent was replaced with a placebo powder	16, 5/11, 30.7 years (No SD and no BMI)	8, 3/5, 29.3 years (No SD and BMI)	SCORAD and Skindex-29-J	IgE, TARC, QoL
Gobel 2010 [88]	Denmark	*Lactobacillus acidophilus* NCFM (1 × 10^10^ CFU/day) and *Bifidobacterium animalis* subsp. (1 × 10^10^ CFU/day)	Probiotic	8 weeks	oral (capsules)	placebo consisted of filler consisting of cellulose, silicon dioxide and rice maltodextrin	for BI-07: 17, 9/8, 18 months and for NCFM: 17, 15/2, 18 months	16, 8/8, 18 months	SCORAD	Total IgE, specific IgE, ECP, fecal calprotectin and the cytokines IL-10, IFN- and IL-31
Liu 2025 [56]	China	15 FMT capsules per week for 3 weeks	FMT	3 weeks	Oral (capsules)	placebo present	20, 10/10, 36.7, BMI N/A	10, 4/6, 36.9, BMI N/A	Proportion achieving EASI 50	SCORAD, IGA, ADCT, DLQI, VAS, POEM, HADS, Aes, reduced Th2/Th17 cell proportions, TNF-α, total IgE, IL-12p70, NK-cell perforin levels
Anania 2025 [51]	Italy	*Bifidobacterium bifidum* PRL2010 (1 × 10^9^ CFU)	Probiotic (single-strain)	From 36 weeks gestation through 6 months postpartum	Oral	Placebo sachets containing maltodextrins, administered on the same schedule as the probiotic	37 mothers, all female, mean age 32.53 ± 5.10 years, BMI not reported	34 mothers, all female, mean age 33.56 ± 4.92 years, BMI not reported	Incidence of atopic dermatitis in infants	SCORAD

**Table 2:** Study characteristics and outcomes of nutraceutical interventions on psoriasis patients including author, country, intervention (strain/dose), type, duration, route, control/placebo, participant demographics, and primary/secondary outcomes.

Author	Country	Intervention + Dose	Type of Intervention	Intervention Duration	Route of Intervention	Control/Placebo	Intervention Patient Demographics (n, M/F, Mean Age (SD), BMI)	Controls Patient Demographics (n, M/F, Mean Age (SD), BMI)	Primary Outcome	Secondary Outcome
Gilli 2023 [91]	Brazil	1 capsule of *Lactobacillus rhamnosus* Lr-G14, 5 billion CFU/g, 1 ×/day	Probiotic (*Lactobacillus rhamnosus*)	60 days	Oral	Placebo capsule	18, 4/14, 51.67 years (14.88), 30.42 (6.656)	17, 10/7, 53.29 (14.59), 29.71 (5.525)	PASI, BSA, and DLQI	IL-17 and IL-23 levels, adverse events
Akbarzadeh 2022 [40]	Iran	Lactocare^®^ probiotic (two times daily) associated with topical hydrocortisone	Synbiotic	12 weeks	Oral	Placebo with topical hydrocortisone	25, 16/9, 44.16 ± 2.18 years, BMI: Not reported	27, 17/10, 38.25 ± 1.79 years, BMI: Not reported	PASI and VAS scores	DLQI scores
Moludi 2021 [89]	Iran	Multi-strain probiotic capsule (*Lactobacillus acidophilus*, *Bifidobacterium bifidum*, *Bifidobacterium lactis*, *Bifidobacterium langum*); 1.8 × 10^9^ CFU, twice daily	Probiotic	8 weeks	Oral	Maltodextrin capsules	25, 15/10, 42.7 ± 9.1 years, BMI: Not reported	25, 17/8, 43.1 ± 7.8 years, BMI: Not reported	PASI	DLQI, Cytokine Levels (IL-6, hs-CRP, MDA)
Drouin 2008 [90]	Canada	XP-828L (Dermylex) 800 mg daily	Probiotic (whey protein extract)	56 days	Oral	Placebo (400 mg tablet of microcrystalline cellulose)	16, 11/5, 45.3 ± 13.9 years, PASI (Baseline): 7.5 ± 1.9, BMI: Not reported	10, 5/5, 55.2 ± 13.9 years, PASI (Baseline): 9.7 ± 3.7, BMI: Not reported	PASI	Itching Sensation and DLQI
Suriano 2023 [55]	Brazil	*Lactobacillus rhamnosus* formula (6 × 10^5^ bacteria/mL), administered as 5 mL daily in a 5% whey solution	Probiotic	6 months	Oral (liquid formula)	Identical whey formula without active probiotics	50 participants, 27/23, mean age 50 years (range 20–76), mean BMI 29.8 (SD = 4.9)	53 participants, 26/27, mean age 52 years (range 18–77), mean BMI 28.3 (SD = 5.7)	PASI	DLQI

**Table 3:** Study characteristics and outcomes of nutraceutical interventions on acne vulgaris patients including author, country, intervention (strain/dose), type, duration, route, control/placebo, participant demographics, and primary/secondary outcomes.

Author	Country	Intervention + Dose	Type of Intervention	Intervention Duration	Route of Intervention	Control/Placebo	Intervention Patient Demographics (n, M/F, Mean Age (SD), BMI)	Controls Patient Demographics (n, M/F, Mean Age (SD), BMI)	Primary Outcome	Secondary Outcome
Liang 2024 [41]	China	*Lactobacillus plantarum* MH-301 (2 × 10^9^ CFU/day)	Probiotic and isotretnoin	12 weeks	Oral	Isotretinoin only	35, 21/14, 22.44 ± 3.59 years, and Probiotic group: 35, 17/18, 21.72 ± 3.54 years	35, 17/18, 22.68 ± 2.78 years, BMI: Not reported	Total lesion count (TLC)	Skin Microbiota Diversity and Gut Microbiota
Eguren 2024 [93]	Spain	*Lacticaseibacillus rhamnosus* CECT 30031 and *Arthrospira platensis* (1 × 10^9^ CFU/day)	Probiotic	12 weeks	Oral	Placebo (maltodextrin capsule)	42, 32/10, 20.13 years, BMI: Not reported	39, 24/15, 18.03 years, BMI: Not reported	Acne Global Severity Scale (AGSS)	Inflammatory Lesions Count, Total Lesion Count, Global Acne Grading System (GAGS)
Kim 2021 [18]	South Korea	CJLP55 1.0 × 10^10^ CFU	Probiotic	12 weeks	Oral	Mixture of maltodextrin and glucose anhydrocrystalline	14, 7/7, 24.29 years ± 0.73, 20.74 ± 0.64	14, 5/9, 23.86 years ± 0.80, 21.39 ± 0.55	Total lesion count (TLC)	Skin sebum, hydration and pH values
Atefi 2025 [42]	Iran	Two daily probiotic capsules from LactoCare (Zist Takhmir, Iran). Each capsule contains seven probiotic strains: *Lactobacillus casei*, *Lactobacillus acidophilus*, *Lactobacillus rhamnosus*, *Lactobacillus bulgaricus*, *Bifidobacterium breve*, *Bifidobacterium longum*, and *Streptococcus thermophilus*, with a colony count exceeding 109 colony-forming units.	Probiotic	8 weeks	Oral	Daily antibacterial face wash and Adapalene gel every other night and one pill of doxycycline per day	40, 20/20, age N/A, 23.67 kg/m^2^	40, 23/17, age N/A, 24.11	Global Acne Grading System (GAGS)	Global Acne Assessment Scale (GAAS), Acne Grading Method
Draelos 2025 [92]	USA	Probiotic (*B. subtilis* DE111) Postbiotic (*L. plantarum* L-137)	Probiotic + postbiotic + other compounds	12 weeks	Oral	inactive ingredients, which included organic rice hulls, cellulose, artificial color, and brown rice flour	47, sex N/A, 30.8, BMI N/A	45, sex N/A, 31.6, BMI N/A	Investigator Global Assessment (IGA)	Inflammatory lesion counts, non-inflammatory lesion counts, blinded investigator skin assessments

**Table 4:** Study characteristics and outcomes of nutraceutical interventions on chronic urticaria patients including author, country, intervention (strain/dose), type, duration, route, control/placebo, participant demographics, and primary/secondary outcomes.

Author	Country	Intervention + Dose	Type of Intervention	Intervention Duration	Route of Intervention	Control/Placebo	Intervention Patient Demographics (n, M/F, Mean Age (SD), BMI)	Controls Patient Demographics (n, M/F, Mean Age (SD), BMI)	Primary Outcome	Secondary Outcome
Dabbaghzadeh 2023 [94]	Iran	Probiotic (Femilact capsule containing *Lactobacillus casei*, *Lactobacillus acidophilus*, *Lactobacillus bulgaricus*, *Lactobacillus rhamnosus*, *Bifidobacterium breve*, *Bifidobacterium longum*) twice daily (2 × 10^9^ CFU/day) + cetirizine	Probiotic	8 weeks	Oral	Placebo	20, 16/4, 24.5 ± 6.7 years, 24.1 ± 5.7	18, 15/3, 27.1 ± 1.7 years, 27.0 ± 5.7	UAS7	Quality of Life (DLQI)
Atefi 2022 [52]	Iran	Synbiotic called LactoCare containing *Lactobacillus rhamnosus*, *Lactobacillus casei*, *Lactobacillus acidophilus*, *Bifidobacterium breve*, *Lactobacillus bulgaricus*, *Bifidobacterium longum*, and *Streptococcus thermophilus*, plus fructooligosaccharides. Administered twice daily in capsule form alongside an antihistamine regimen	Synbiotic	8 weeks	Oral capsules	Antihistamines	21 patients, aged 18–45 years, mean age 36.50 years (SD = 10.75)	21 patients, mean age 40.21 years (SD = 13.74)	Urticaria Activity Score (UAS7)	DLQI, Severity Categories and Response Rates and Symptom Reduction

**Table 5:** Study characteristics and outcomes of nutraceutical interventions on melasma patients including author, country, intervention (strain/dose), type, duration, route, control/placebo, participant demographics, and primary/secondary outcomes.

Author	Country	Intervention + Dose	Type of Intervention	Intervention Duration	Route of Intervention	Control/Placebo	Intervention Patient Demographics (n, M/F, Mean Age (SD), BMI)	Controls Patient Demographics (n, M/F, Mean Age (SD), BMI)	Primary Outcome	Secondary Outcome
Piyavatin 2021 [95]	Thailand	Synbiotic TS6 Supplement (containing six probiotic strains and prebiotics) administered at 1 sachet daily	Synbiotic (Probiotics + Prebiotics)	12 weeks	Oral (sachets)	Placebo with identical properties but without the active synbiotics/prebiotics	29, 0/29 39.45 ± 7.02 years, BMI: Not reported	28, 0/25, 41.38 ± 7.59 years, BMI: Not reported	mMASI	Melanin and Erythema Index

## Data Availability

The original contributions presented in the study are included in the article/Supplementary Materials, further inquiries can be directed to the corresponding author.

## Supplementary Materials

The following supporting information can be downloaded at: https://www.mdpi.com/article/10.3390/microorganisms14010063/s1, PRISMA 2020 checklist.

## Abbreviations

AD	Atopic Dermatitis
AGSS	Acne Global Severity Scale
AhR	Aryl Hydrocarbon Receptor
CDLQI	Children’s Dermatology Life Quality Index
DLQI	Dermatology Life Quality Index
FMT	Fecal Microbiota Transplantation
FOS	Fructooligosaccharide
FXR	Farnesoid X Receptor
GALT	Gut-Associated Lymphoid Tissue
GOS	Galactooligosaccharide
GPCR	G-Protein-Coupled Receptor
HDAC	Histone Deacetylase
IDQoL	Infant Dermatology Quality of Life Index
IQR	Interquartile Range
LPS	Lipopolysaccharide
mMASI	Modified Melasma Area and Severity Index
MHC	Major Histocompatibility Complex
PASI	Psoriasis Area and Severity Index
PAMP	Pathogen-Associated Molecular Pattern
PRISMA	Preferred Reporting Items for Systematic Reviews and Meta-Analyses
QoL	Quality of Life
RCT	Randomized Controlled Trial
RoB 2	Risk of Bias Version 2 Tool
SCFAs	Short-Chain Fatty Acids
SCORAD	Scoring Atopic Dermatitis Index
TARC	Thymus and Activation-Regulated Chemokine
TLR	Toll-Like Receptor
TLC	Total Lesion Count
TNF-α	Tumor Necrosis Factor Alpha
Treg	Regulatory T Cell
TSLP	Thymic Stromal Lymphopoietin
UAS7	Urticaria Activity Score Over 7 Days
WAO	World Allergy Organization

## References

[1] Seth D., Cheldize K., Brown D., Freeman E.E. (2017). Global Burden of Skin Disease: Inequities and Innovations. Curr. Derm. Rep..

[2] Parisi R., Iskandar I.Y.K., Kontopantelis E., Augustin M., Griffiths C.E.M., Ashcroft D.M. (2020). National, Regional, and Worldwide Epidemiology of Psoriasis: Systematic Analysis and Modelling Study. BMJ.

[3] Pu Y., He L., Wang X., Zhang Y., Zhao S., Fan J. (2024). Global, Regional, and National Levels and Trends in Burden of Urticaria: A Systematic Analysis for the Global Burden of Disease Study 2019. J. Glob. Health.

[4] Wang L.-J., Pang Y.-B., Li W.-Q., He Q.-Y., Zhang X.-E., Liu E., Guo J. (2024). Global Research Trends on Melasma: A Bibliometric and Visualized Study from 2014 to 2023. Front. Pharmacol..

[5] Lolou V., Panayiotidis M.I. (2019). Functional Role of Probiotics and Prebiotics on Skin Health and Disease. Fermentation.

[6] Wakelin S. (2013). Dermatological Pharmacology: Systemic Drugs. Medicine.

[7] Ellis S.R., Nguyen M., Vaughn A.R., Notay M., Burney W.A., Sandhu S., Sivamani R.K. (2019). The Skin and Gut Microbiome and Its Role in Common Dermatologic Conditions. Microorganisms.

[8] Zhao M., Chu J., Feng S., Guo C., Xue B., He K., Li L. (2023). Immunological Mechanisms of Inflammatory Diseases Caused by Gut Microbiota Dysbiosis: A Review. Biomed. Pharmacother..

[9] Valdes A.M., Walter J., Segal E., Spector T.D. (2018). Role of the Gut Microbiota in Nutrition and Health. BMJ.

[10] Mahmud M.R., Akter S., Tamanna S.K., Mazumder L., Esti I.Z., Banerjee S., Akter S., Hasan R., Acharjee M., Hossain S. (2022). Impact of Gut Microbiome on Skin Health: Gut-Skin Axis Observed through the Lenses of Therapeutics and Skin Diseases. Gut Microbes.

[11] De Pessemier B., Grine L., Debaere M., Maes A., Paetzold B., Callewaert C. (2021). Gut–Skin Axis: Current Knowledge of the Interrelationship between Microbial Dysbiosis and Skin Conditions. Microorganisms.

[12] Lee A. (2015). Recent Progress in Melasma Pathogenesis. Pigment Cell Melanoma Res..

[13] Passos M.D.C.F., Moraes-Filho J.P. (2017). Intestinal microbiota in digestive diseases. Arq. Gastroenterol..

[14] O’Neill C.A., Monteleone G., McLaughlin J.T., Paus R. (2016). The Gut-skin Axis in Health and Disease: A Paradigm with Therapeutic Implications. BioEssays.

[15] Zhang L., Cao H., Li L., Zhao W., Zhang F. (2022). Oral and External Intervention on the Crosstalk between Microbial Barrier and Skin via Foodborne Functional Component. J. Funct. Foods.

[16] Huang L., Gao R., Yu N., Zhu Y., Ding Y., Qin H. (2019). Dysbiosis of Gut Microbiota Was Closely Associated with Psoriasis. Sci. China Life Sci..

[17] Alam M.J., Xie L., Yap Y.-A., Marques F.Z., Robert R. (2022). Manipulating Microbiota to Treat Atopic Dermatitis: Functions and Therapies. Pathogens.

[18] Kim M.-J., Kim K.-P., Choi E., Yim J.-H., Choi C., Yun H.-S., Ahn H.-Y., Oh J.-Y., Cho Y. (2021). Effects of *Lactobacillus plantarum* CJLP55 on Clinical Improvement, Skin Condition and Urine Bacterial Extracellular Vesicles in Patients with Acne Vulgaris: A Randomized, Double-Blind, Placebo-Controlled Study. Nutrients.

[19] Liu R., Peng C., Jing D., Xiao Y., Zhu W., Zhao S., Zhang J., Chen X., Li J. (2021). Biomarkers of Gut Microbiota in Chronic Spontaneous Urticaria and Symptomatic Dermographism. Front. Cell. Infect. Microbiol..

[20] Rezazadeh A., Shahabi S., Bagheri M., Nabizadeh E., Jazani N.H. (2018). The Protective Effect of *Lactobacillus* and *Bifidobacterium* as the Gut Microbiota Members against Chronic Urticaria. Int. Immunopharmacol..

[21] Liu Y., Du X., Zhai S., Tang X., Liu C., Li W. (2022). Gut Microbiota and Atopic Dermatitis in Children: A Scoping Review. BMC Pediatr..

[22] Wu K., Lee T.-H., Chen Y.-L., Wang Y.-S., Wang P.-H., Yu C.-P., Chu K.-H., Chiang Y.-R. (2019). Metabolites Involved in Aerobic Degradation of the A and B Rings of Estrogen. Appl. Environ. Microbiol..

[23] Yu C.-P., Deeb R.A., Chu K.-H. (2013). Microbial Degradation of Steroidal Estrogens. Chemosphere.

[24] Maguire M., Maguire G. (2017). The Role of Microbiota, and Probiotics and Prebiotics in Skin Health. Arch. Dermatol. Res..

[25] Eslami M., Bahar A., Keikha M., Karbalaei M., Kobyliak N.M., Yousefi B. (2020). Probiotics Function and Modulation of the Immune System in Allergic Diseases. Allergol. Immunopathol..

[26] Gore C., Custovic A., Tannock G.W., Munro K., Kerry G., Johnson K., Peterson C., Morris J., Chaloner C., Murray C.S. (2012). Treatment and Secondary Prevention Effects of the Probiotics *Lactobacillus paracasei* or *Bifidobacterium lactis* on Early Infant Eczema: Randomized Controlled Trial with Follow-up until Age 3 Years. Clin. Exp. Allergy.

[27] Ma T., Shen X., Shi X., Sakandar H.A., Quan K., Li Y., Jin H., Kwok L.-Y., Zhang H., Sun Z. (2023). Targeting Gut Microbiota and Metabolism as the Major Probiotic Mechanism—An Evidence-Based Review. Trends Food Sci. Technol..

[28] Gowda V., Sarkar R., Verma D., Das A. (2024). Probiotics in Dermatology: An Evidence-Based Approach. Indian Dermatol. Online J..

[29] Notay M., Foolad N., Vaughn A.R., Sivamani R.K. (2017). Probiotics, Prebiotics, and Synbiotics for the Treatment and Prevention of Adult Dermatological Diseases. Am. J. Clin. Dermatol..

[30] Polak K., Jobbágy A., Muszyński T., Wojciechowska K., Frątczak A., Bánvölgyi A., Bergler-Czop B., Kiss N. (2021). Microbiome Modulation as a Therapeutic Approach in Chronic Skin Diseases. Biomedicines.

[31] Zou X., Zou X., Gao L., Zhao H. (2024). Gut Microbiota and Psoriasis: Pathogenesis, Targeted Therapy, and Future Directions. Front. Cell. Infect. Microbiol..

[32] Fanfaret I., Boda D., Ion L., Hosseyni D., Leru P., Ali S., Corcea S., Bumbacea R. (2021). Probiotics and Prebiotics in Atopic Dermatitis: Pros and Cons (Review). Exp. Ther. Med..

[33] Prado Franco De Godoy L., Garcia Michalichen F., Cartagena Rossi R., Bortoletto Gomes De Aquino S., Ferreira F.R. (2023). Use of Oral Probiotics in Inflammatory Skin Diseases. Literature Review. Port. J. Dermatol. Venereol..

[34] Umborowati M.A., Damayanti D., Anggraeni S., Endaryanto A., Surono I.S., Effendy I., Prakoeswa C.R.S. (2022). The Role of Probiotics in the Treatment of Adult Atopic Dermatitis: A Meta-Analysis of Randomized Controlled Trials. J. Health Popul. Nutr..

[35] Zeng L., Yu G., Wu Y., Hao W., Chen H. (2021). The Effectiveness and Safety of Probiotic Supplements for Psoriasis: A Systematic Review and Meta-Analysis of Randomized Controlled Trials and Preclinical Trials. J. Immunol. Res..

[36] Page M.J., Moher D., Bossuyt P.M., Boutron I., Hoffmann T.C., Mulrow C.D., Shamseer L., Tetzlaff J.M., Akl E.A., Brennan S.E. (2021). PRISMA 2020 Explanation and Elaboration: Updated Guidance and Exemplars for Reporting Systematic Reviews. BMJ.

[37] Kim J.E., Kim H.S. (2019). Microbiome of the Skin and Gut in Atopic Dermatitis (AD): Understanding the Pathophysiology and Finding Novel Management Strategies. JCM.

[38] Carucci L., Nocerino R., Paparo L., De Filippis F., Coppola S., Giglio V., Cozzolino T., Valentino V., Sequino G., Bedogni G. (2022). Therapeutic Effects Elicited by the Probiotic *Lacticaseibacillus rhamnosus* GG in Children with Atopic Dermatitis. The Results of the ProPAD Trial. Pediatr. Allergy Immunol..

[39] Prakoeswa C.R.S., Bonita L., Karim A., Herwanto N., Umborowati M.A., Setyaningrum T., Hidayati A.N., Surono I.S. (2022). Beneficial Effect of *Lactobacillus plantarum* IS-10506 Supplementation in Adults with Atopic Dermatitis: A Randomized Controlled Trial. J. Dermatol. Treat..

[40] Akbarzadeh A., Alirezaei P., Doosti-Irani A., Mehrpooya M., Nouri F. (2022). The Efficacy of Lactocare^®^ Synbiotic on the Clinical Symptoms in Patients with Psoriasis: A Randomized, Double-Blind, Placebo-Controlled Clinical Trial. Dermatol. Res. Pract..

[41] Liang L., Qi X., Jiang X., Chen T., Dong L. (2024). *Lactobacillus plantarum* MH-301 as an Effective Adjuvant to Isotretinoin in the Treatment of Acne Vulgaris: A Randomized and Open-Label Trail. Front. Med..

[42] Atefi N., Mohammadi M., Bodaghabadi M., Mehrali M., Behrangi E., Ghassemi M., Jafarzadeh A., Goodarzi A. (2025). Evaluating the Effectiveness of Probiotic Supplementation in Combination with Doxycycline for the Treatment of Moderate Acne: A Randomized Double-Blind Controlled Clinical Trial. J. Cosmet. Dermatol..

[43] Cukrowska B., Ceregra A., Maciorkowska E., Surowska B., Zegadło-Mylik M.A., Konopka E., Trojanowska I., Zakrzewska M., Bierła J.B., Zakrzewski M. (2021). The Effectiveness of Probiotic *Lactobacillus rhamnosus* and *Lactobacillus casei* Strains in Children with Atopic Dermatitis and Cow’s Milk Protein Allergy: A Multicenter, Randomized, Double Blind, Placebo Controlled Study. Nutrients.

[44] Nakata J., Hirota T., Umemura H., Nakagawa T., Kando N., Futamura M., Nakamura Y., Ito K. (2019). Additive Effect of Lactobacillus Acidophilus L-92 on Children with Atopic Dermatitis Concomitant with Food Allergy. Asia Pac. Allergy.

[45] Drago L., Iemoli E., Rodighiero V., Nicola L., De Vecchi E., Piconi S. (2011). Effects of *Lactobacillus salivarius* LS01 (DSM 22775) Treatment on Adult Atopic Dermatitis: A Randomized Placebo-Controlled Study. Int. J. Immunopathol. Pharmacol..

[46] Fölster-Holst R., Müller F., Schnopp N., Abeck D., Kreiselmaier I., Lenz T., Von Rüden U., Schrezenmeir J., Christophers E., Weichenthal M. (2006). Prospective, Randomized Controlled Trial on *Lactobacillus rhamnosus* in Infants with Moderate to Severe Atopic Dermatitis. Br. J. Dermatol..

[47] Brouwer M.L., Wolt-Plompen S.A.A., Dubois A.E.J., Van Der Heide S., Jansen D.F., Hoijer M.A., Kauffman H.F., Duiverman E.J. (2006). No Effects of Probiotics on Atopic Dermatitis in Infancy: A Randomized Placebo-controlled Trial. Clin. Exp. Allergy.

[48] Rather I.A., Kim B.-C., Lew L.-C., Cha S.-K., Lee J.H., Nam G.-J., Majumder R., Lim J., Lim S.-K., Seo Y.-J. (2021). Oral Administration of Live and Dead Cells of *Lactobacillus sakei* proBio65 Alleviated Atopic Dermatitis in Children and Adolescents: A Randomized, Double-Blind, and Placebo-Controlled Study. Probiotics Antimicro. Prot..

[49] Nermes M., Kantele J.M., Atosuo T.J., Salminen S., Isolauri E. (2011). Interaction of Orally Administered *Lactobacillus rhamnosus* GG with Skin and Gut Microbiota and Humoral Immunity in Infants with Atopic Dermatitis. Clin. Exp. Allergy.

[50] Yoshida Y., Seki T., Matsunaka H., Watanabe T., Shindo M., Yamada N., Yamamoto O. (2010). Clinical Effects of Probiotic *Bifidobacterium breve* Supplementation in Adult Patients with Atopic Dermatitis. Yonago Acta Medica.

[51] Anania C., Matys V., Marra S., De Canditiis D., Olivero F., Carraro C., Giugliano A., Zicari A.M., Piccioni M.G. (2025). Effect of Supplementation with a Specific Probiotic (*Bifidobacterium bifidum* PRL2010) in Pregnancy for the Prevention of Atopic Dermatitis in Children: Preliminary Results of a Randomized Trial. Nutrients.

[52] Atefi N., Fallahpour M., Sharifi S., Ghassemi M., Roohaninasab M., Goodarzi A. (2022). Probiotic as an Adjuvant Therapy in Chronic Urticaria: A Blinded Randomized Controlled Clinical Trial. Eur. Ann. Allergy Clin. Immunol..

[53] Lin R.-J., Qiu L.-H., Guan R.-Z., Hu S.-J., Liu Y.-Y., Wang G.-J. (2015). Protective Effect of Probiotics in the Treatment of Infantile Eczema. Exp. Ther. Med..

[54] Farid R., Ahanchian H., Jabbari F., Moghiman T. (2011). Effect of a New Synbiotic Mixture on Atopic Dermatitis in Children: A Randomized-Controlled Trial. Iran. J. Pediatr..

[55] Suriano E.S., Souza M.D.M., Kobata C.M., Santos F.H.Y., Mimica M.J. (2023). Efficacy of an Adjuvant *Lactobacillus rhamnosus* Formula in Improving Skin Lesions as Assessed by PASI in Patients with Plaque Psoriasis from a University-Affiliated, Tertiary-Referral Hospital in São Paulo (Brazil): A Parallel, Double-Blind, Randomized Clinical Trial. Arch. Dermatol. Res..

[56] Liu X., Luo Y., Chen X., Wu M., Xu X., Tian J., Gao Y., Zhu J., Wang Z., Zhou Y. (2025). Fecal Microbiota Transplantation against Moderate-to-severe Atopic Dermatitis: A Randomized, Double-blind Controlled Explorer Trial. Allergy.

[57] Shafiei A., Moin M., Pourpak Z., Gharagozlou M., Aghamohammadi A., Sajedi V., Soheili H., Sotoodeh S., Movahedi M. (2011). Synbiotics Could Not Reduce the Scoring of Childhood Atopic Dermatitis (SCORAD): A Randomized Double Blind Placebo-Controlled Trial. Iran. J. Allergy Asthma Immunol..

[58] Van Der Aa L.B., Heymans H.S., Van Aalderen W.M., Sillevis Smitt J.H., Knol J., Ben Amor K., Goossens D.A., Sprikkelman A.B., the Synbad Study Group (2010). Effect of a New Synbiotic Mixture on Atopic Dermatitis in Infants: A Randomized-controlled Trial. Clin. Exp. Allergy.

[59] Wu Y.-J., Wu W.-F., Hung C.-W., Ku M.-S., Liao P.-F., Sun H.-L., Lu K.-H., Sheu J.-N., Lue K.-H. (2017). Evaluation of Efficacy and Safety of *Lactobacillus rhamnosus* in Children Aged 4–48 Months with Atopic Dermatitis: An 8-Week, Double-Blind, Randomized, Placebo-Controlled Study. J. Microbiol. Immunol. Infect..

[60] Boženský J., Hill M., Zelenka R., Skýba T. (2015). Prebiotics Do Not Influence the Severity of Atopic Dermatitis in Infants: A Randomised Controlled Trial. PLoS ONE.

[61] Prakoeswa C.R.S., Herwanto N., Prameswari R., Astari L., Sawitri S., Hidayati A.N., Indramaya D.M., Kusumowidagdo E.R., Surono I.S. (2017). *Lactobacillus plantarum* IS-10506 Supplementation Reduced SCORAD in Children with Atopic Dermatitis. Benef. Microbes.

[62] D’Auria E., Panelli S., Lunardon L., Pajoro M., Paradiso L., Beretta S., Loretelli C., Tosi D., Perini M., Bedogni G. (2021). Rice Flour Fermented with *Lactobacillus paracasei* CBA L74 in the Treatment of Atopic Dermatitis in Infants: A Randomized, Double-Blind, Placebo-Controlled Trial. Pharmacol. Res..

[63] Michelotti A., Cestone E., De Ponti I., Giardina S., Pisati M., Spartà E., Tursi F. (2021). Efficacy of a Probiotic Supplement in Patients with Atopic Dermatitis: A Randomized, Double-Blind, Placebo-Controlled Clinical Trial. Eur. J. Dermatol..

[64] Yamamoto K., Yokoyama K., Matsukawa T., Kato S., Kato S., Yamada K., Hirota T. (2016). Efficacy of Prolonged Ingestion of *Lactobacillus acidophilus* L-92 in Adult Patients with Atopic Dermatitis. J. Dairy. Sci..

[65] Shibata R., Kimura M., Takahashi H., Mikami K., Aiba Y., Takeda H., Koga Y. (2009). Clinical Effects of Kestose, a Prebiotic Oligosaccharide, on the Treatment of Atopic Dermatitis in Infants. Clin. Exp. Allergy.

[66] Yan D.-C., Hung C.-H., Sy L.B., Lue K.-H., Shih I.-H., Yang C.-Y., Chen L.-C., Sun H.-L., Lee M.-S., Chambard J. (2019). A Randomized, Double-Blind, Placebo-Controlled Trial Assessing the Oral Administration of a Heat-Treated *Lactobacillus paracasei* Supplement in Infants with Atopic Dermatitis Receiving Topical Corticosteroid Therapy. Ski. Pharmacol. Physiol..

[67] Woo S.-I., Kim J.-Y., Lee Y.-J., Kim N.-S., Hahn Y.-S. (2010). Effect of Lactobacillus Sakei Supplementation in Children with Atopic Eczema–Dermatitis Syndrome. Ann. Allergy Asthma Immunol..

[68] Feíto-Rodríguez M., Ramírez-Boscà A., Vidal-Asensi S., Fernández-Nieto D., Ros-Cervera G., Alonso-Usero V., Prieto-Merino D., Núñez-Delegido E., Ruzafa-Costas B., Sánchez-Pellicer P. (2023). Randomized Double-Blind Placebo-Controlled Clinical Trial to Evaluate the Effect of a Mixture of Probiotic Strains on Symptom Severity and Use of Corticosteroids in Children and Adolescents with Atopic Dermatitis. Clin. Exp. Dermatol..

[69] Inoue Y., Kambara T., Murata N., Komori-Yamaguchi J., Matsukura S., Takahashi Y., Ikezawa Z., Aihara M. (2014). Effects of Oral Administration of *Lactobacillus acidophilus* L-92 on the Symptoms and Serum Cytokines of Atopic Dermatitis in Japanese Adults: A Double-Blind, Randomized, Clinical Trial. Int. Arch. Allergy Immunol..

[70] Chernyshov P.V. (2009). Randomized, Placebo-Controlled Trial on Clinical and Immunologic Effects of Probiotic Containing *Lactobacillus rhamnosus* R0011 and *L. Helveticus* R0052 in Infants with Atopic Dermatitis. Microb. Ecol. Health Dis..

[71] Ahn S.H., Yoon W., Lee S.Y., Shin H.S., Lim M.Y., Nam Y.-D., Yoo Y. (2020). Effects of *Lactobacillus pentosus* in Children with Allergen-Sensitized Atopic Dermatitis. J. Korean Med. Sci..

[72] Yeşilova Y., Çalka Ö., Akdeniz N., Berktaş M. (2012). Effect of Probiotics on the Treatment of Children with Atopic Dermatitis. Ann. Dermatol..

[73] Isolauri E., Arvola T., SÜtas Y., Moilanen E., Salminen S. (2000). Probiotics in the Management of Atopic Eczema. Clin. Exp. Allergy.

[74] Wang L., Wang F., Gershwin M.E. (2015). Human Autoimmune Diseases: A Comprehensive Update. J. Intern. Med..

[75] Sharma R., Handa S., Mahajan R., De D., Sachdeva N. (2022). Evaluating the Effect of Supplementation with *Bacillus clausii* on Therapeutic Outcomes in Atopic Eczema—Results of an Observer-Blinded Parallel-Group Randomized Controlled Study. Indian. J. Dermatol..

[76] Rosenfeldt V., Benfeldt E., Nielsen S.D., Michaelsen K.F., Jeppesen D.L., Valerius N.H., Paerregaard A. (2003). Effect of Probiotic Lactobacillus Strains in Children with Atopic Dermatitis. J. Allergy Clin. Immunol..

[77] Iemoli E., Trabattoni D., Parisotto S., Borgonovo L., Toscano M., Rizzardini G., Clerici M., Ricci E., Fusi A., De Vecchi E. (2012). Probiotics Reduce Gut Microbial Translocation and Improve Adult Atopic Dermatitis. J. Clin. Gastroenterol..

[78] Andrade P.D.S.M.A.d., Maria e Silva J., Carregaro V., Sacramento L.A., Roberti L.R., Aragon D.C., Carmona F., Roxo-Junior P. (2022). Efficacy of Probiotics in Children and Adolescents with Atopic Dermatitis: A Randomized, Double-Blind, Placebo-Controlled Study. Front. Nutr..

[79] Prescott S.L., Dunstan J.A., Hale J., Breckler L., Lehmann H., Weston S., Richmond P. (2005). Clinical Effects of Probiotics Are Associated with Increased Interferon-γ Responses in Very Young Children with Atopic Dermatitis. Clin. Exp. Allergy.

[80] Kirjavainen P.V., Salminen S.J., Isolauri E. (2003). Probiotic Bacteria in the Management of Atopic Disease: Underscoring the Importance of Viability. J. Pediatr. Gastroenterol. Nutr..

[81] Jeong S., Huang L.-K., Tsai M.-J., Liao Y.-T., Lin Y.-S., Hu C.-J., Hsu Y.-H. (2022). Cognitive Function Associated with Gut Microbial Abundance in Sucrose and S-Adenosyl-L-Methionine (SAMe) Metabolic Pathways. J. Alzheimer’s Dis..

[82] Navarro-López V., Ramírez-Boscá A., Ramón-Vidal D., Ruzafa-Costas B., Genovés-Martínez S., Chenoll-Cuadros E., Carrión-Gutiérrez M., Horga De La Parte J., Prieto-Merino D., Codoñer-Cortés F.M. (2018). Effect of Oral Administration of a Mixture of Probiotic Strains on SCORAD Index and Use of Topical Steroids in Young Patients with Moderate Atopic Dermatitis: A Randomized Clinical Trial. JAMA Dermatol..

[83] Gerasimov S.V., Vasjuta V.V., Myhovych O.O., Bondarchuk L.I. (2010). Probiotic Supplement Reduces Atopic Dermatitis in Preschool Children: A Randomized, Double-Blind, Placebo-Controlled, Clinical Trial. Am. J. Clin. Dermatol..

[84] Viljanen M., Savilahti E., Haahtela T., Juntunen-Backman K., Korpela R., Poussa T., Tuure T., Kuitunen M. (2005). Probiotics in the Treatment of Atopic Eczema/Dermatitis Syndrome in Infants: A Double-blind Placebo-controlled Trial. Allergy.

[85] Grüber C., Wendt M., Sulser C., Lau S., Kulig M., Wahn U., Werfel T., Niggemann B. (2007). Randomized, Placebo-controlled Trial of *Lactobacillus rhamnosus* GG as Treatment of Atopic Dermatitis in Infancy. Allergy.

[86] Han Y., Kim B., Ban J., Lee J., Kim B.J., Choi B.S., Hwang S., Ahn K., Kim J. (2012). A Randomized Trial of *Lactobacillus plantarum* CJLP133 for the Treatment of Atopic Dermatitis. Pediatr. Allergy Immunol..

[87] Weston S. (2005). Effects of Probiotics on Atopic Dermatitis: A Randomised Controlled Trial. Arch. Dis. Child..

[88] Gobel R.J., Larsen N.N., Mølgaard C., Jakobsen M., Michaelsen K.F. (2010). Probiotics to Young Children with Atopic Dermatitis: A Randomized Placebo-Controlled Trial. Int. J. Probiotics Prebiotics.

[89] Moludi J., Khedmatgozar H., Saiedi S., Razmi H., Alizadeh M., Ebrahimi B. (2021). Probiotic Supplementation Improves Clinical Outcomes and Quality of Life Indicators in Patients with Plaque Psoriasis: A Randomized Double-Blind Clinical Trial. Clin. Nutr. ESPEN.

[90] Drouin R., Moroni O., Cantin K., Juneau C. (2008). A Double-Blind, Placebo-Controlled, Randomized Trial of XP-828L (800 Mg) on the Quality of Life and Clinical Symptoms of Patients with Mild-to-Moderate Psoriasis. Altern. Med. Rev..

[91] Gilli I.O., Da Silva G.C., Mendes V., Duarte M.G., Tanaka A.A. (2023). The Role of Probiotics as an Adjunctive Therapy in Psoriasis. J. Psoriasis Psoriatic Arthritis.

[92] Draelos Z., Harper J., Farris P.K., Hazan A., Raymond I. (2025). A 12-Week Randomized, Double-Blind, Placebo-Controlled Trial for the Efficacy and Safety of a Novel Nutraceutical for Mild-to-Moderate Acne. J. Cosmet. Dermatol..

[93] Eguren C., Navarro-Blasco A., Corral-Forteza M., Reolid-Pérez A., Setó-Torrent N., García-Navarro A., Prieto-Merino D., Núñez-Delegido E., Sánchez-Pellicer P., Navarro-López V. (2024). A Randomized Clinical Trial to Evaluate the Efficacy of an Oral Probiotic in Acne Vulgaris. Acta Derm. Venereol..

[94] Dabbaghzadeh A., Ghaffari J., Moradi S., Sayadian separghan D. (2023). Probiotics on Chronic Urticaria: A Randomized Clinical Trial Study. Casp. J. Intern. Med..

[95] Piyavatin P., Chaichalotornkul S., Nararatwanchai T., Bumrungpert A., Saiwichai T. (2021). Synbiotics Supplement Is Effective for Melasma Improvement. J. Cosmet. Dermatol..

[96] Chouraqui J.-P. (2021). Does the Contribution of Human Milk Oligosaccharides to the Beneficial Effects of Breast Milk Allow Us to Hope for an Improvement in Infant Formulas?. Crit. Rev. Food Sci. Nutr..

[97] Du B., Shama A., Zhang Y., Chen B., Bu Y., Chen P., Lin C., Liu J., Zheng J., Li Z. (2025). Gut Microbiota and Plasma Metabolites in Pregnant Mothers and Infant Atopic Dermatitis: A Multi-Omics Study. World Allergy Organ. J..

[98] Reddel S., Del Chierico F., Quagliariello A., Giancristoforo S., Vernocchi P., Russo A., Fiocchi A., Rossi P., Putignani L., El Hachem M. (2019). Gut Microbiota Profile in Children Affected by Atopic Dermatitis and Evaluation of Intestinal Persistence of a Probiotic Mixture. Sci. Rep..

[99] Xiao X., Hu X., Yao J., Cao W., Zou Z., Wang L., Qin H., Zhong D., Li Y., Xue P. (2023). The Role of Short-Chain Fatty Acids in Inflammatory Skin Diseases. Front. Microbiol..

[100] Trompette A., Pernot J., Perdijk O., Alqahtani R.A.A., Domingo J.S., Camacho-Muñoz D., Wong N.C., Kendall A.C., Wiederkehr A., Nicod L.P. (2022). Gut-Derived Short-Chain Fatty Acids Modulate Skin Barrier Integrity by Promoting Keratinocyte Metabolism and Differentiation. Mucosal Immunol..

[101] Akbarzadeh A., Taheri M., Ebrahimi B., Alirezaei P., Doosti-Irani A., Soleimani M., Nouri F. (2022). Evaluation of Lactocare^®^ Synbiotic Administration on the Serum Electrolytes and Trace Elements Levels in Psoriasis Patients: A Randomized, Double-Blind, Placebo-Controlled Clinical Trial Study. Biol. Trace Elem. Res..

[102] Kober M.-M., Bowe W.P. (2015). The Effect of Probiotics on Immune Regulation, Acne, and Photoaging. Int. J. Women’s Dermatol..

[103] Deng Y., Wang H., Zhou J., Mou Y., Wang G., Xiong X. (2018). Patients with Acne Vulgaris Have a Distinct Gut Microbiota in Comparison with Healthy Controls. Acta Derm. Venerol..

[104] Wang A., Rojas O., Lee D., Gommerman J.L. (2021). Regulation of Neuroinflammation by B Cells and Plasma Cells. Immunol. Rev..

[105] Zhao M., Shen C., Ma L. (2018). Treatment Efficacy of Probiotics on Atopic Dermatitis, Zooming in on Infants: A Systematic Review and Meta-analysis. Int. J. Dermatol..

[106] Wu Y., Wang X., Wu W., Yang J. (2024). Mendelian Randomization Analysis Reveals an Independent Causal Relationship between Four Gut Microbes and Acne Vulgaris. Front. Microbiol..

[107] Proctor L.M., Creasy H.H., Fettweis J.M., Lloyd-Price J., Mahurkar A., Zhou W., Buck G.A., Snyder M.P., Strauss J.F., The Integrative HMP (iHMP) Research Network Consortium (2019). The Integrative Human Microbiome Project. Nature.

